# Treatment of Landfill Leachate Using an Advanced Microwave Reactor Coupled with a Batch and Continuous Algal Photobioreactor

**DOI:** 10.1021/acsomega.5c13308

**Published:** 2026-03-18

**Authors:** Binay Kumar Tripathy, Ranjeet Kumar Mishra, Mathava Kumar

**Affiliations:** † Manipal Institute of Technology, 76793 Manipal Academy of Higher Education, Manipal 576104, India; ‡ Environmenal and Water Resources Engineering Division, Department of Civil Engineering, Indian Institute of Technology Madras, Chennai, Tamil Nadu 600039, India

## Abstract

Landfill leachate poses a significant environmental challenge due to its high concentrations of organic pollutants, nutrients, and toxic metals. This study presents a hybrid microwave-coagulation-algal (M-C-A) photobioreactor system that operates in batch and continuous-flow modes for effective leachate treatment. The hybrid system integrates microwave-assisted removal, coagulation, and algal bioremediation to enhance pollutant removal efficiency. Furthermore, the microwave pretreatment achieved 83.6% ammonia removal at 95 °C, thereby reducing leachate toxicity and enhancing the subsequent biological treatment. Coagulation using FeCl_3_ further removed 76% of the COD and 90% of the turbidity. The pretreated leachate was further subjected to algal photobioreactor treatment, during which optimal growth occurred at a 50% leachate dilution, resulting in 77% total nitrogen (TN) removal and 17% total phosphorus (TP) removal. In the continuous-flow algal sequencing batch reactor (ASBR), the maximum TN and TP removal rates were 23.50 and 2.66 g/m^3^/d, respectively. The heavy metals Zn^2+^ and Pb^2+^ were removed, with Fe removal reaching up to 92%. The harvested algal biomass exhibited a calorific value of 16.50 MJ/kg, indicating its potential for biofuel production. Finally, the integrated M-C-A system demonstrated efficient removal of organic matter, nutrients, and metals, while enabling biomass valorization. The continuous flow operation ensures scalability and operational stability, making it a promising sustainable technology for managing landfill leachate and recovering resources.

## Graphical abstract

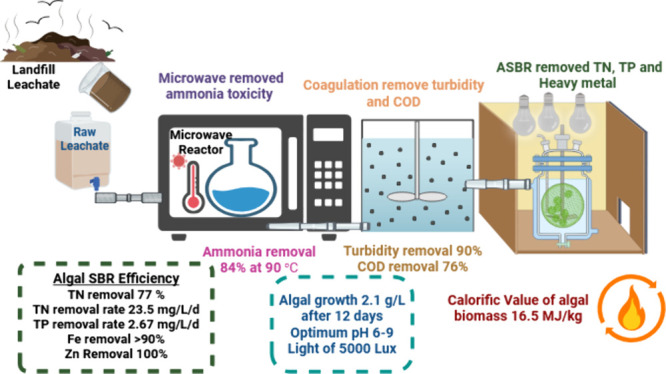

## 1 Introduction

The rapid pace of urbanization, industrialization, and population growth over the past few decades has dramatically increased global municipal solid waste (MSW) generation. According to the World Bank, global solid waste production was approximately 2.24 billion tons per year in 2020 and is projected to reach nearly 3.88 billion tons annually by 2050 if current trends continue.^1^ The management of this escalating waste load poses a formidable environmental and socio-economic challenge. Landfilling remains the most adopted waste management strategy worldwide due to its low cost and operational simplicity.^2^ However, it represents a major environmental liability due to the generation of landfill leachate, a complex, highly polluted liquid formed by the percolation of rainwater and the biochemical degradation of waste materials within landfill cells. Conventional leachate treatment systems typically employ a combination of physicochemical and biological methods. Physicochemical techniques, including coagulation-flocculation, precipitation, adsorption, ion exchange, membrane filtration, and advanced oxidation processes (AOPs), have demonstrated effectiveness in removing suspended solids, turbidity, and recalcitrant organic pollutants.^3,4^ The membrane-based technologies, such as reverse osmosis or nanofiltration, though efficient, face fouling problems and require regular maintenance.^5^ The biological treatments, such as sequencing batch reactors (SBRs), membrane bioreactors (MBRs), and upflow anaerobic sludge blanket (UASB) reactors, are cost-effective and environmentally safe methods for leachate treatment.^6^ The biological treatment is often limited by the toxicity of landfill leachate, particularly the high concentrations of ammonia, heavy metals, and refractory organics that inhibit microbial growth and enzymatic activity.^7^ Furthermore, biological systems typically require long retention times and are sensitive to fluctuations in influent characteristics, temperature, and pH.^7^

To overcome the limitations of standalone methods, hybrid treatment technologies that integrate physicochemical and biological processes have gained increasing attention in recent years. Microwave irradiation offers unique advantages such as rapid volumetric heating, selective energy transfer to polar molecules, and generation of localized “hot spots” that facilitate the breakdown of complex organic compounds and ammonia.^8^ Algae-based treatment systems have recently emerged as sustainable biological alternatives for nutrient recovery and biomass valorization. Algal growth yields biomass that can be harvested and converted into value-added products, including biodiesel, biochar, and biofertilizers.^9^

Algal photobioreactors (PBRs) are flexible in operation and can be integrated into hybrid systems for the simultaneous removal of pollutants and production of bioenergy. However, direct application of landfill leachate in algal systems remains challenging due to high ammonia concentrations, metal toxicity, and low light penetration.^10^ These factors can inhibit photosynthesis, chlorophyll synthesis, and algal metabolism.^11^ Furthermore, combining microwave and coagulation pretreatments before algal cultivation offers a promising approach to overcoming these limitations by reducing the toxic load and improving nutrient bioavailability. The integration of microwave, coagulation, and algal photobioreactor processes into a single hybrid system represents a novel and synergistic approach to treating landfill leachate.^11^ Microalgae utilize residual nutrients (nitrogen and phosphorus) in the algal system, contributing to the removal of organics and metal content while generating biomass that can be valorized as a bioenergy feedstock.^9^ Figure 1a illustrates the yearly publication trends on a particular research topic indexed in Google Scholar and Scopus from 2015 to 2026. The search was made using the phrase “microwave reactor for landfill leachate treatment” on Google Scholar and the Scopus database on 27/10/2025.

**Figure 1:**
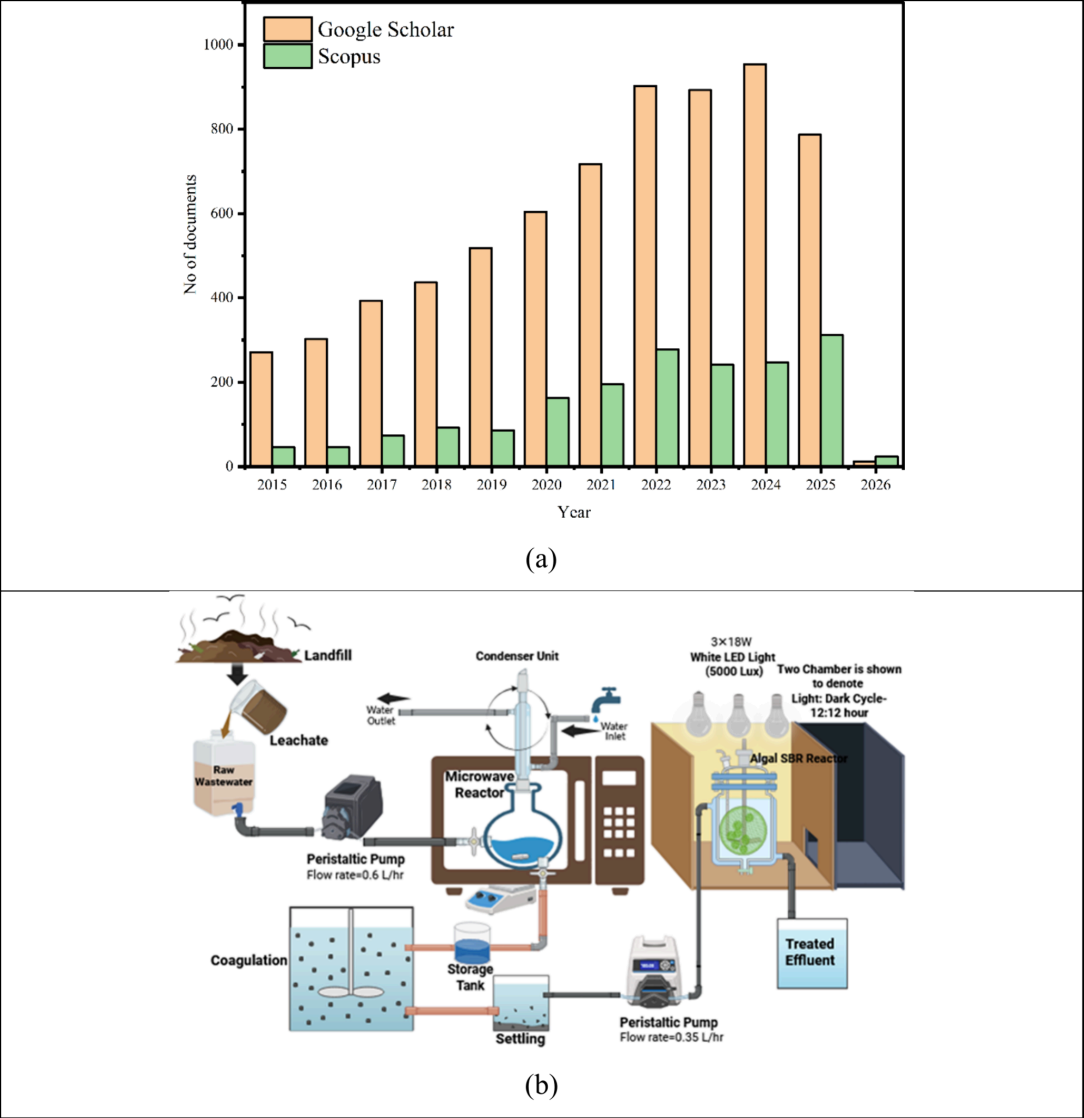
(a) Number of documents available on google scholar and scopus database on microwave reactor for landfill leachate treatment, and (b) schematic of the continuous M (Cont.)-C-ASBR system for leachate treatment.

Several studies have reported on landfill leachate treatment using microwave and algae-based technologies individually. Yeh et al. investigated landfill leachate treatment using microwave oxidation, employing the Taguchi method.^12^ They reported that 80% TOC removal and 96% color removal were achieved at 550 W, 1 M persulfate, and 120 min. Similarly, the use of iron–carbon and persulfate under MW irradiation resulted in 94.56% TOC removal from landfill leachate at a MW power of 240 W, a reaction time of 10 min, and optimal oxidant dosages (Fe–C of 1 g/L and PS of 30 mM).^13^ Meanwhile, coagulation treatment using alum and polyaluminum chloride (PAC) followed by microwave persulfate oxidation was studied for landfill leachate, and the coagulant and persulfate dosage were optimized using a Box-Behnken design.^14^ The combined treatment achieved 79.20% COD and 91.2% UV_254_ from the landfill leachate. A combination of the MW-activated peroxyacetic process and a semiaerobic aged refuse biofilter was used to oxidize the effluent from MBR-treated landfill leachate.^15^ MW oxidation removed 80% of the TOC and 42.24% of the UV_254_, which enhanced nitrification in the biofilter. The MW oxidation reduced the refractory and toxic organic compounds in the leachate, thereby improving bacterial community growth in the subsequent biological process and increasing nitrogen removal rates. Furthermore, the algal tubular photobioreactor was developed to remove nitrogen (NO_3_–N and NO_2_–N) from landfill leachate using algal species, such as *Chlorella vulgaris* and *Tetradesmus obliquus*. It was observed that maximum algal biomass productivity occurred using *Chlorella vulgaris* at 7.50% leachate dilution, and 100% NO_3_–N removal was observed at 5% leachate dilution (21 mg/L NO_3_–N) with an 11:1 N:P ratio.^16^ Similarly, a coculture of algal species, such as *Chlorella vulgaris* and *Scenedesmus dimorphus* (1:1 w/w), was used for landfill leachate pretreated with NaClO, followed by dilution (5–15%) to optimize algal growth.^17^ NaClO pretreatment removed 49% of the COD and 52% of the total nitrogen (TN) at initial concentrations of 14,000 and 4400 mg/L, respectively. The secondary algal process removed 81% of COD, 72.10% of TN, and 86% of TP after 10 days at a 10% leachate concentration, 4000 Lux illumination, and a light-to-dark ratio of 12:12 h. Furthermore, a combination of coagulation using FeCl_3_, alum, and polyaluminum chloride (PAC), along with an algal treatment using algae collected and enriched from the fish pond, was employed for landfill leachate treatment.^18^ The combined treatment achieved removal of 63–72.40% COD, 75% BOD, 87.5–93.4% NH_4_–N and 73.6–86.7% PO_4_–P from leachate (initial ammonia concentration of 894–1377 mg/L). After carefully reviewing the literature, it was noted that most studies focused on the individual use of microwave technology and algae-based treatments. However, the authors noted that the best available knowledge is that the combined use of a microwave reactor and an alga-based photobioreactor is absent in the literature. Few studies have explored the synergistic interactions among microwave-assisted removal, chemical coagulation, and algal metabolism in a continuous-flow configuration, especially under real leachate variability. The influence of microwave parameters, such as power intensity, exposure time, and frequency, on subsequent algal nutrient uptake and metal bioaccumulation remains poorly understood to date.

Therefore, to address the aforementioned research gap, the present study designed a hybrid reactor comprising a microwave (M (Cont.) and an algal photobioreactor (ASBR) for treating landfill leachate. The microwave reactor was operated in continuous flow mode at 500 W, 0.6 L/h, and 95 °C. Moreover, coagulation pretreatment was employed in conjunction with microwave treatment, and the treated sample was then fed into the algal photobioreactor. The hybrid reactor was operated in continuous flow mode, with an algal photobioreactor operated as an SBR at an HRT of 3 days, light illumination of 5000 Lux (white LED), and a flow rate of 0.35 L/h. The effects of microwave and coagulation pretreatment on the subsequent algal process were investigated by measuring COD, alkalinity, total nitrogen content (TN), total phosphate (TP), and heavy metals. The present study aligned with the United Nations Sustainable Development Goals (SDG 6: Clean Water and Sanitation, SDG 12: Responsible Consumption and Production, SDG 13: Climate Action, SDG 14: Life Below Water, and SDG 15: Life on Land).

## 2 Materials and Methods

### 2.1 Leachate Collection and Algal Strain Culture

The landfill leachate sample used in this study was collected from the Perungudi dump yard, located in Chennai, India (Latitude: 12°57′13.5″ N; Longitude: 80°14′5.8″ E). All necessary safety measures and standard protocols were strictly followed during sample collection, transportation, and handling to prevent contamination and ensure representative sampling. The collected leachate was immediately transferred into airtight, high-density polyethylene containers and stored at 4 °C to preserve its physicochemical integrity until further analysis and experimentation. In parallel, the native algal strain was isolated from the same Perungudi landfill site to improve its adaptability to the leachate matrix. The isolated microalgae were cultured and maintained in Bold’s Basal Medium (BBM) under controlled laboratory conditions (white LED light of 5000 Lx, temperature of 25 °C, mixing rate of 300 rpm during the batch and continuous study) using the algal photobioreactor setup. The culture was periodically acclimatized and subcultured to maintain exponential growth prior to use in batch and continuous flow experiments. The algae collected from the landfill site were initially grown with 100% BBM for 10 days and then fed 5% leachate to acclimate to leachate toxicity. The 5% leachate was selected based on the published literature, ammonia, and alkalinity toxicity.

### 2.2 Chemicals

Analytical-grade sulfuric acid (H_2_SO_4_, 99% purity) and sodium hydroxide (NaOH) were procured from Merck, India, while ferric chloride (FeCl_3_) was obtained from Rankem Pvt. Ltd., India. All chemicals and reagents used in this study were of analytical grade and were employed without further purification.

### 2.3 Microwave Reactor and Algal Photobioreactor

Figure 1b illustrates an integrated treatment process for landfill leachate and raw wastewater, utilizing a combination of physicochemical and biological methods to achieve efficient pollutant removal. Initially, the leachate is collected and pumped through a peristaltic pump (flow rate: 0.6 L/h) into a microwave reactor (Ragatech Pvt. Ltd.) equipped with a condenser unit for thermal treatment, which enhances the breakdown of complex organic compounds. The microwave was operated at 0–1000 W and a frequency of 2450 Hz (constant microwave supply and frequency). Additionally, the microwave power can be regulated through an integrated timer, ensuring precise control over the treatment process. The setup includes a magnetic stirrer positioned at the base to maintain uniform mixing, a 1 L reactor vessel for sample processing, and a water recirculation system to sustain consistent cooling and operational efficiency. The treated effluent from the reactor undergoes coagulation and settling to remove suspended solids and colloidal matter, followed by storage for subsequent processing. The clarified water is transferred using another peristaltic pump (flow rate: 0.35 L/h) to an algal sequencing batch reactor (SBR) illuminated with white LED lights (5000 Lux) under a controlled 12:12 h light-dark cycle. At this stage, microalgae facilitate the uptake of nutrients and the further biodegradation of residual organic matter, as well as nitrogen and phosphorus compounds. The integrated approach combines microwave-assisted removal with biological polishing, yielding enhanced leachate detoxification and improved effluent quality. The overall system effectively minimizes chemical oxygen demand (COD), total suspended solids (TSS), and nutrient levels, demonstrating a promising hybrid technology for eco-friendly treatment and resource recovery from landfill leachate and municipal wastewater streams.

### 2.4 Coagulation and Microwave Sequential Experiment

The coagulation experiments were performed using a standard jar test apparatus, with ferric chloride (FeCl_3_) employed as the coagulant in accordance with Standard Methods (APHA, 2012). The optimum coagulant dosage was determined to be 1 g/L at a pH of 5.5. Prior to the coagulation step, a 1:1 dilution of the leachate with distilled water was carried out to minimize foaming, which can interfere with proper sludge settling.^19^ This dilution also reduced ammonia toxicity, thereby improving algal growth performance in subsequent SBR experiments. Further, the coagulation trials were conducted before and after microwave (M) treatment to identify the optimal sequence and coagulant dosage. During microwave treatment, the leachate sample was irradiated at 500 W for 10 min throughout all experiments. For the C-M configuration, 500 mL of leachate was first coagulated, and the supernatant obtained after the sludge settled was collected and stored. Subsequently, 100 mL of the coagulated leachate was transferred into a 1 L microwave reactor vessel for treatment. The optimal microwave exposure time of 10 min was selected based on the ammonia removal efficiency observed in preliminary batch trials. Similarly, in the M-C configuration, 100 mL of raw leachate was first treated in the microwave reactor for 10 min and then subjected to coagulation. The treated samples were collected and stored until sufficient volume was accumulated for further experiments. The diluted sample (1:1 prior to coagulation) was designated MD-C and used in subsequent coagulation and algal SBR studies. All dilutions were performed using distilled water to maintain consistency across experiments.

### 2.5 Sequential Microwave Coagulation and Algal Study (MD-C-Algal)

The preliminary algal cultivation and batch studies were conducted using treated leachate samples, which were diluted with Bold’s Basal Medium (BBM) to provide essential nutrients for algal growth. The detailed composition and preparation procedure of BBM are presented in Table 1. The algal photobioreactor was operated under a controlled light-dark cycle of 12:12 h, with an illumination intensity of approximately 5000 Lux provided by white LED lamps (Figure 1b). At the start of each experiment, 100 mL of the treated leachate sample was inoculated with a precultured algal suspension, and the system was monitored for 12 consecutive days. The leachate concentration varied from 10 to 100% (100% corresponding to undiluted leachate) by adjusting the BBM-to-treated sample ratio. The effects of leachate concentration and pH on algal biomass growth, biomass productivity, and total nitrogen (TN) removal were systematically investigated.

**Table 1:** Physicochemical Properties of the MD-C Sample

parameter	raw	C (raw)	C (1:1 dilution)	MD-C
pH	7.8	3	3.8	4
turbidity (NTU)	127	13	3	3
color	8000	1100	400	380
alkalinity (mg/L as CaCO_3_)	15,000			200
COD (mg/L)	4000	960	450	400
BOD (mg/L)	120	65	60	20
BOD/COD	0.03	0.06	0.03	0.05
Cl-(mg/L)	1600	2300	2000	2050
sulfate (mg/L)	55	1500	980	1000
NH_3_–N(mg/L)	2000	1750	950	100
TN (as mg/L of N)	2397	1800	1100	120
Fe-Total (mg/L)	14	40	30	25
zinc (mg/L)	0.9	0.6	0.3	0.325
copper (mg/L)	4	2.2	2	1.8
manganese (mg/L)	4	2.2	1.2	1.4
lead(mg/L)	1.2	0.17	0.08	0.084

### 2.6 MD-C-ASBR (Batch) and MD-C-ASBR (Cont.) Study

The leachate sample obtained after the MD-C experiment was stored and subsequently introduced into the algal sequencing batch reactor (SBR) using a peristaltic pump operating at a flow rate of 0.35 L/h, based on a 50% leachate concentration (designated as MD-C-ASBR). The dilution was carried out using algal culture previously grown in Bold’s Basal Medium (BBM) to ensure optimal inoculation and nutrient balance within the reactor. The SBR was operated for a total duration of 15 days, comprising a filling time of 30 min, a reaction period of 3 days, a settling phase of 4 h, and a decanting time of 30 min per cycle. At the end of each 3-day cycle, the reactor was replenished with a fresh batch of MD-C-treated leachate. Daily routine monitoring of key physicochemical parameters, including pH, alkalinity, chemical oxygen demand (COD), dissolved oxygen (DO), and total nitrogen (TN), was performed. Total phosphorus (TP) and metal concentrations were analyzed at the completion of each cycle.

The microwave reactor (M-reactor) was operated in continuous-flow mode at 95 °C, with the leachate sample introduced at a flow rate of 0.6 L/h using a peristaltic pump. Microwave heating was maintained for 1 h to achieve effective thermal treatment. The pretreated effluent from the microwave reactor was subsequently diluted (1:1) and subjected to coagulation using FeCl_3_ at an optimized dosage of 0.8 g/L (denoted as MD-C (cont.)). The resulting effluent was then supplied to the algal SBR (MD-C-ASBR (cont.)) for further biological treatment. During continuous operation, periodic sampling was performed to evaluate the removal of organic matter, nitrogen, phosphorus, and heavy metals, providing insights into the overall performance and stability of the integrated hybrid system.

## 3 Results and Discussions

### 3.1 Ammonia Removal in Microwave Treatment

The influence of temperature and flow rate on the performance of the microwave (M) reactor was systematically evaluated during continuous-flow leachate treatment. As illustrated in Figure 2a, the effect of temperature on ammonia removal was examined at a constant flow rate of 0.6 L/h. An optimum ammonia removal efficiency of 83.6% was achieved at 95 °C, resulting in a final ammonia concentration of 360 mg/L. In contrast, variations in flow rate between 0.4 and 0.8 L/h resulted in only marginal changes in ammonia removal efficiency (Figure 2b). These findings indicate that temperature exerts a more dominant influence on ammonia volatilization than flow rate under the given operating conditions. Furthermore, the previous studies have also demonstrated similar trends in continuous microwave-assisted systems. For instance, microwave-UV photolysis used for the degradation of monochloroacetic acid (MCAA) at higher flow rates (5–15 L/h) exhibited a decline in removal efficiency with increasing flow rate, primarily due to reduced contact time and lower energy exposure per unit volume.^20^ The selected flow rate of <1 L/h ensured sufficient residence time to facilitate uniform microwave heating and enhance ammonia removal, which is particularly critical for complex leachate matrices with high organic and nitrogen loads. Comparable observations were reported in a pilot-scale investigation on coke wastewater treatment using a microwave-alone process, where ammonia removal decreased from 96% in batch operation to approximately 80% under continuous flow at a feed rate of 5 m^3^/d and a microwave power of 4.8 kW.^21^ The consistency of these results across different systems reinforces the importance of optimizing flow rate and temperature to achieve efficient microwave-assisted ammonia removal in continuous reactor configurations. Ammonia volatilization usually occurs primarily through stripping at elevated pH and temperature. However, raising pH to 11–12 requires high chemical and operational costs. Moreover, ammonia released into the atmosphere has serious air pollution and regulatory compliance issues. However, the present study explores pH 8–8.5 for the ammonia removal. Mahmoud et al. reported 100% ammonia removal at low ammonia concentration over pH 8.2–8.35. The mechanism of removal was attributed to ionic conductivity and dipole relaxation time.^22^ Frequent dipole vibrations induced by MW nonionizing radiation can weaken the hydrogen bonds between N–H· · ·O and O–H· · ·N in the sample. This facilitates the escape of ammonia even at lower pH, with high efficiency and in a very short time. Although microwaves can have thermal and athermal effects on different reactions, the microwave thermal effects were noted to be predominant during ammonia removal. Furthermore, the microwave oxidation in the presence of nickel-based catalysts and mesoporous carbon achieved 99% conversion of ammonia to hydrogen.^23^

**Figure 2:**
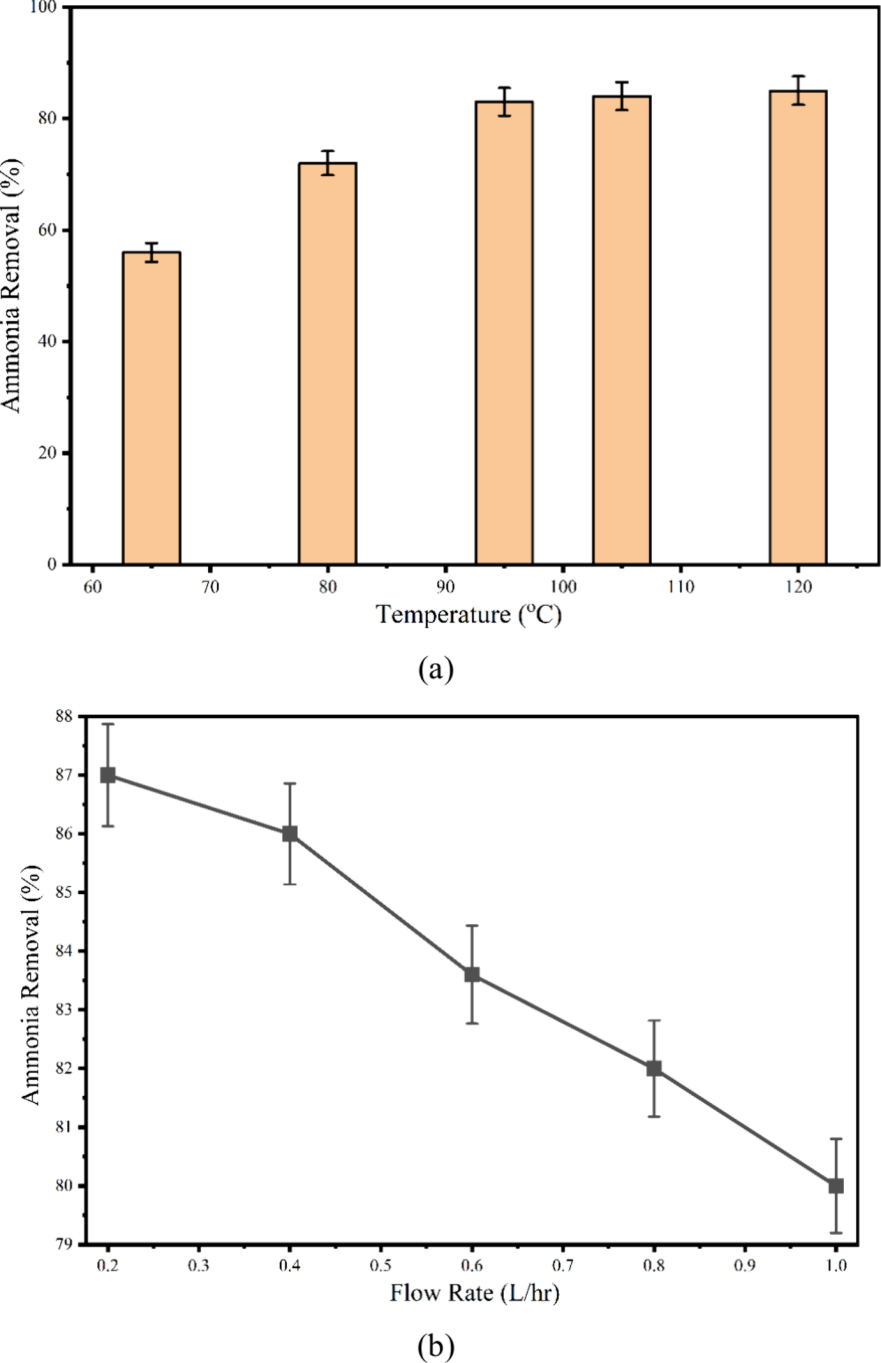
Effect of (a) temperature and (b) flow rate on ammonia removal in M-C continuous reactor.

### 3.2 Turbidity and COD Removal in Coagulation Pretreatment

Substantial reductions in turbidity and COD were observed during the coagulation (C-treatment) process, primarily due to the destabilization and aggregation of suspended colloidal and organic matter by FeCl_3_.^24^ The choice of FeCl_3_ is based on maximum removal of organics and TP from landfill leachate through sweep flocculation at neutral pH as observed from the literature. For instance, Swar et al. used different types of coagulants such as alum, FeCl_3_ and poly aluminum chloride (PAC) before the algal reactor for leachate treatment, and FeCl_3_ resulted in maximum COD, NH_4_–N and PO_4_–P removal (36, 57, and 56%, respectively).^18^ Further, Fe^2+^ is considered necessary for algal growth and excess Fe^2+^ is consumed by algal cells through luxury uptake. Approximately 90% turbidity removal and 76% COD reduction were achieved at an optimal FeCl_3_ dosage of 1.60 g/L, indicating efficient coagulation and floc formation. The coagulation sludge collected (20–25% V/V) was dried to analyze the composition of the sludge using EDAX in Table S1 and Figure S1a of the supplementary file. The C content of 28.8% and the Fe content of 10.5% shows sweep flocculation of organics through Fe­(OH)_3_ flocs. Further, the SEM image shown in Figure S1b indicates that the sludge contain microporous structure with Fe deposition. The dried sludge can be used for the preparation of magnetic sludge-derived biochar for the removal of tertiary pollutants from wastewater.^25^ The hydrolysis of FeCl_3_ in water can result in the formation of Fe­(OH)_3,_ leading to sweep flocculation and removal of pollutants.^26^ However, this treatment alone was ineffective in removing ammonia, as coagulation predominantly targets particulate and nonionic organic contaminants rather than dissolved nitrogenous compounds. Conversely, microwave treatment (M-treatment) did not achieve significant turbidity reduction, likely because it mainly facilitates thermal degradation and partial oxidation of organic molecules rather than particulate separation.^20^ Hence, integrating microwave and coagulation was found to be essential for comprehensive leachate purification. The microwave step helps break down complex organics and improve leachate biodegradability, while subsequent coagulation effectively removes suspended solids and residual COD. In the combined processes, FeCl_3_ dosage was optimized based on treatment sequence and dilution conditions: 0.8 g/L for postmicrowave coagulation with 1:1 dilution (MD-C and MD-C (cont.)), 1.6 g/L for C-M treatment, and 1.4 g/L for M-C treatment. These optimized dosages ensured efficient pollutant removal while minimizing chemical consumption. The detailed physicochemical characteristics of the leachate after coagulation under these conditions, specifically for the MD-C and MD-C (cont.) samples, are presented in Table 2.

**Table 2:** Characteristics of Sample Fed into MD-C-ASBR (Cont.) System

parameters	raw leachate	M (Cont.)	MD-C (Cont.)
pH	7.8	9	4
turbidity (NTU)	127	130	3
COD (mg L^–1^)	4000	3800	400
alkalinity (mg L^–1^ as CaCO_3_)	15,000	14,400	200
TN (mg L^–1^)	2397	400	200
NH_3_–N(mg L^–1^)	2000	360	180
TP (mg L^–1^as PO_4_^3–^)	36	38	12
C: N:P ratio	133:80:1	100:10:1	33:16:1
zinc (mg L^–1^)	0.9	0.87	0.3
copper (mg L^–1^)	0.194	0.19	1.2
manganese (mg L^–1^)	1.8	1.7	0.7
lead (mg L^–1^)	0.8	0.8	0.14
Fe total (mg L^–1^)	14	13	28

### 3.3 Algal Batch Study Experiment

The pretreated leachate samples obtained from various treatment sequences, namely raw leachate, coagulation alone (C-alone), microwave alone (M-alone), coagulation followed by microwave (C-M), and microwave followed by coagulation (M-C), were subsequently introduced into an algal batch reactor to assess biomass growth and nutrient uptake performance. The temporal variation in algal biomass concentration for each treatment condition is illustrated in Figure 3a. It was observed that the variation in algal biomass growth, as represented by fluorescence intensity (RFU), over 12 days for different pretreated leachate samples Raw, C-alone, M-alone, C-M, M-C, and MD-C, along with a Control. Figure S2a in the supplementary file shows the concentrations of ammonia and COD in each unit of the coupled MD-C-algal process. It indicates that COD and ammonia have been efficiently removed from the leachate. Figure S2b shows the pH variation across different unit processes. The algal growth pattern demonstrates a clear influence of pretreatment on biomass productivity. The control culture exhibited steady growth, reaching a maximum fluorescence of approximately 110 RFU by day 10, which served as the reference for optimal algal performance. In contrast, the raw leachate showed the lowest growth (below 50 RFU), attributed to high pollutant loads, turbidity, and ammonia toxicity, which inhibited photosynthetic activity. Chang et al. employed a membrane-based algal photobioreactor using *C. vulgaris* to remove color and ammonia in the membrane module, resulting in elevated algal growth of 2.13 g/L after 12 days.^27^ The study observed that landfill leachate without membrane pretreatment reduced the light intensity from 9102 to 2129 Lux due to its color, significant turbidity, and suspended solids. The C-alone and C-M treatments showed moderate improvements, indicating partial removal of organic and particulate matter that improved light penetration but did not sufficiently reduce ammonia concentration. M-alone treatment achieved better growth (80 RFU), likely due to partial organic degradation and increased nutrient bioavailability after microwave exposure. Remarkably, the MD-C treatment exhibited the highest algal growth (135 RFU), surpassing even the control, suggesting that dilution and sequential microwave-coagulation treatment effectively optimized nutrient balance (especially N:P ratio), reduced toxicity, and improved water clarity, creating highly favorable conditions for algal proliferation. The M-C treatment also showed enhanced ammonia removal and moderate growth, confirming the importance of treatment order and pH effects on nutrient dynamics. Wang et al. found that the major inhibitory factors for mixed algae composed of *Chlorella and Microcystis sp.* in landfill leachate were chromaticity, free ammonia nitrogen (FAN), and molecular organic matter (MOM), with chromaticity having the greatest inhibitory effect, followed by MOM and FAN.^28^

**Figure 3:**
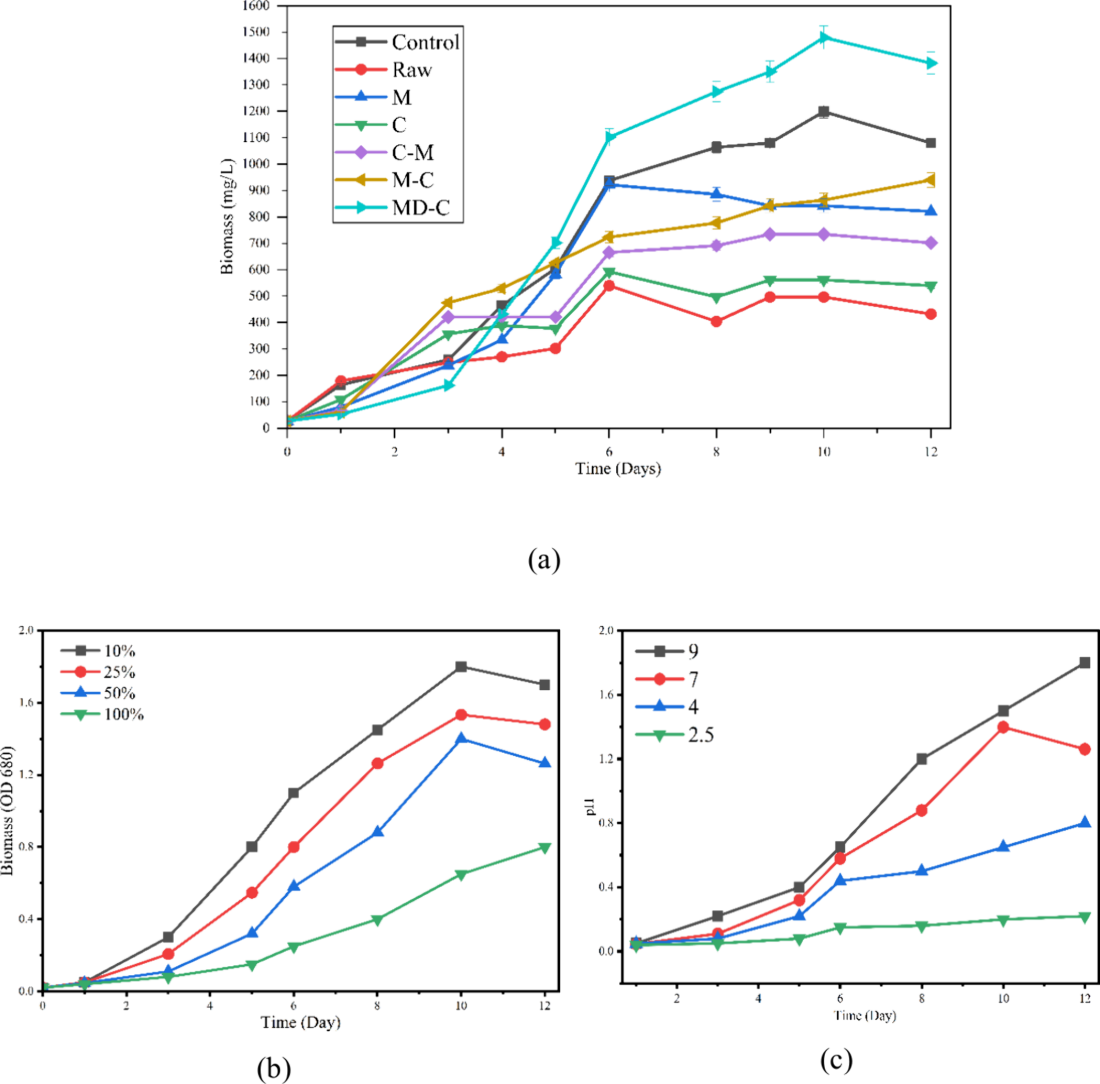
(a) Growth of algal biomass in a batch-scale algal reactor with different pretreated samples. (b) Effect of leachate concentration (at pH 7) and (c) pH (50% leachate conc.) on algal growth in the MD-C algal system; initial algal biomass of 50 mg L^–1^, light intensity of 5000 Lux.

Among the tested conditions, algal growth in the C-M-treated leachate was significantly higher than in the raw leachate, indicating that the combined pretreatment effectively reduced the organic and inorganic pollutant load. The initial coagulation step (C) removed suspended solids and organic matter, while subsequent microwave treatment (M) contributed to the partial oxidation of refractory compounds, increasing nutrient bioavailability for algal assimilation. Interestingly, microwave treatment was applied before coagulation (M-C), resulting in higher ammonia removal efficiency than in the C-M system. This improvement can be attributed to the elevated pH conditions generated during microwave heating, which enhance ammonia volatilization and improve the efficiency of FeCl_3_ coagulation. Previous studies have shown that ammonia removal through both stripping and precipitation mechanisms is favored under alkaline conditions, thereby justifying the superior performance of the M-C configuration. Furthermore, when a 1:1 dilution was performed before the coagulation step following microwave treatment (MD-C), a remarkable enhancement in algal growth was observed. This improvement is primarily due to adjustments in the nutrient balance, particularly the nitrogen-to-phosphorus (N:P) ratio, and to the reduction of potential toxicity in the diluted leachate. The MD-C sample supported the highest algal biomass accumulation, indicating that an optimal combination of microwave-assisted conditioning, dilution, and coagulation significantly improved leachate quality and promoted a nutrient composition more conducive to algal metabolism and growth (Figure 3a)**.** Swar et al. studied the algal-based treatment of landfill leachate pretreated by coagulation-flocculation within a combined physicochemical and algal treatment strategy.^18^ First, an optimized coagulation-flocculation (CF) pretreatment using coagulants such as FeCl_3_, Alum, and PAC was applied to reduce the pollutant load. This was followed by an algal treatment using a mixed microalgal culture. The results demonstrated substantial removal efficiencies: chemical oxygen demand (COD) was reduced by approximately 63–72%, BOD by 75%, ammonium-nitrogen by 88–93%, and phosphate by 74–87%. These outcomes demonstrate that the CF step effectively reduced the major pollutant load, thereby enhancing the performance of the subsequent algal stage, making the combined process a promising alternative for treating mature landfill leachate.^18^

### 3.4 Algal Biomass: Rate of Growth and Productivity

Figure 3b illustrates the variation in algal biomass (optical density at 680 nm) over a 12-day cultivation period at leachate dilution ratios of 10, 25, 50, and 100%. The trend clearly shows that algal growth is highly dependent on leachate dilution, indicating that contaminant and nutrient concentrations strongly influence biomass productivity. At 10% dilution, the culture exhibited the highest biomass accumulation, reaching an OD_6__8_0 of approximately 1.75 by day 10, followed by a slight stabilization, signifying that the nutrient composition and toxicity were within the optimal range for algal metabolism. The 25% dilution also supported substantial growth, achieving an OD_6__8_0 of around 1.5, though slightly lower than the 10% dilution, suggesting the presence of moderately inhibitory substances at higher concentrations. The 50% dilution resulted in a reduced maximum biomass (OD_6__8_0 = 1.3), indicating that the increased organic and ammonia content began to impose stress on the algal cells, limiting photosynthetic efficiency and cell proliferation. In contrast, the 100% (undiluted) leachate exhibited the lowest growth performance, with an OD_6__8_0 of barely 0.8 by day 12. This is likely due to excessive levels of ammoniacal nitrogen, heavy metals, and refractory organic compounds, which are known to inhibit algal enzymatic activity, reduce chlorophyll synthesis, and interfere with light penetration.^27^ The progressive decrease in growth with reduced dilution highlights the importance of controlling leachate concentration prior to biological treatment. Overall, the results demonstrate that proper dilution mitigates the inhibitory effects of toxic compounds while maintaining adequate nutrient availability.

Among all tested conditions, 10–25% dilution provided the most favorable balance for algal growth, suggesting that predilution is a critical step to enhance biomass yield and optimize nutrient uptake efficiency during leachate bioremediation. A 50-fold dilution was employed in a study using an algal photo sequencing batch reactor for treating secondary effluent, aiming to reduce total ammoniacal nitrogen (TAN) to 15 mg/L.^29^ Figure 3c depicts the influence of initial pH on algal biomass growth (measured as OD_6__8_0) during a 12-day cultivation period. Four pH levels (2.5, 4, 7, and 9) were evaluated to determine the optimal pH range for algal proliferation and metabolic activity in leachate-based media. The growth curves clearly show that pH profoundly affects algal performance by regulating nutrient availability, enzymatic activity, and cell membrane stability. At an acidic pH of 2.5, algal growth was minimal throughout the experiment, with an OD_6__8_0 of less than 0.2 even after 12 days. Such lower growth is attributed to the acidic environment, which causes proton stress, impairs photosystem activity, and may lead to metal toxicity.^18^ At pH 4, growth slightly improved but remained suboptimal (OD_6__8_0 = 0.8 at day 12), indicating that mild acidity still hampers photosynthesis and nutrient uptake. Furthermore, under neutral conditions (pH 7), algal growth was significantly enhanced, with biomass reaching an optical density (OD_6__8_0) of approximately 1.3 by day 10 (biomass of 1.54 g/L), followed by a slight plateau. This suggests that neutral pH favors enzymatic reactions, carbon fixation, and chlorophyll synthesis, thereby supporting optimal cell division and nutrient assimilation. However, the highest biomass production was observed at pH 9, with an OD_6__8_0 of approximately 1.8 on day 12 (biomass of 2.1 g/L). The enhanced growth at alkaline pH can be explained by increased ammonia volatilisation, reduced toxicity, and better availability of carbonates, which serve as an additional inorganic carbon source for photosynthesis. The results clearly indicate that algal growth and productivity are strongly dependent on pH. Acidic conditions severely inhibit cell activity, whereas neutral to slightly alkaline environments promote healthy growth and biomass accumulation. Therefore, maintaining the medium at pH 8–9 provides optimal conditions for algal-based leachate treatment, ensuring efficient nutrient utilization and pollutant removal. Lin et al. investigated the use of ammoniacal nitrogen-tolerant microalgae in landfill leachate treatment.^10^ Microalgae (*Chlorella pyrenoidosa* and *Chlamydomonas snowiae*) were used to treat landfill leachate containing 670 mg/L of ammoniacal nitrogen. Both strains exhibited strong tolerance and achieved significant nutrient removal, reducing ammoniacal-N by up to 85%, orthophosphate by 78%, and COD by 65% within 10 days of cultivation. Biomass productivity increased with nutrient uptake, confirming effective nutrient assimilation. Additionally, the phytotoxicity of treated leachate decreased markedly, with Brassica chinensis seed germination improving from 10% (untreated) to nearly 50% after algal treatment. These results demonstrate the potential of ammonia-tolerant microalgae for sustainable leachate bioremediation (Lin et al.). The addition of zeolites has been beneficial in reducing ammonia toxicity to 300 mg/L by adsorbing ammonia and slowly releasing it during algal metabolism during algal treatment of piggery wastewater with high ammonia concentration.^30^ The highest biomass yield (3.25 g/L) was observed, indicating that it was achieved through effective control of ammonia and pH levels. The pH was controlled at 6 to reduce ammonia loss by volatilization and minimize air pollution (by reducing the potential interaction between ammonia molecules and the atmosphere). Furthermore, the ammonia removal was 92% at an initial ammonia concentration of 300 mg/L, compared with 63.50% at 500 mg/L.

### 3.5 Growth Rate and Productivity of Algal Biomass

The experimental results presented in the table demonstrate the performance of an algal treatment system (MD-C-A system) at varying leachate concentrations (10–100%) compared to a control culture grown in a synthetic medium. The parameters analyzed include algal growth rate, biomass productivity, and TN removal efficiency (Table 3). The results indicate that leachate concentration and pretreatment significantly influence algal performance. At a 10% leachate concentration, the system achieved a moderate growth rate of 0.24 d-1 and a productivity of 180 g/m^3^/d, with an excellent TN removal efficiency of 86%. This enhanced performance suggests that at lower concentrations, the nutrient balance and reduced toxicity create favorable conditions for algal proliferation and nitrogen assimilation. The biomass growth after 20 days in an algal SBR using *Chlorella sp.* in synthetic municipal wastewater was found to be 0.77 and 1.08 g/L under natural sunlight and artificial light, respectively.^31^ The biomass productivity was calculated as 93.0–118.60 mg/L/d. Furthermore, at a 20% concentration, the growth rate improved to 0.31 d^–1^, but productivity decreased slightly to 156.10 g/m^3^/d, indicating that while the nutrient supply increased, inhibitory compounds such as ammonia or heavy metals may have begun to affect biomass yield. Selvaratnam et al. studied the algal treatment of landfill leachate with an initial NH_4_–N concentration of 950 mg/L. They reported that the growth of *G. sulphuraria* reached 0.19 g/L/d at a 20% dilution, compared to 0.098 g/L/d at a 40% dilution, and beyond 40% dilution, algal growth was minimal.^32^

**Table 3:** Growth Rate and Algae Productivity in Different Systems in Batch Experiments

sample type	leachate conc. (%)	rate of growth (d^–1^)	productivity (g/m^3^/d)	TN removal efficiency (%)
control	0	0.32 ± 0.02	114 ± 8.0	70 ± 2.5
M-D-C-A system	10	0.24 ± 0.02	180 ± 7.5	86 ± 2.0
	20	0.31 ± 0.01	156.1 ± 5.0	80 ± 2.0
	50	0.34 ± 0.01	132.6 ± 5.0	77 ± 3.0
	100	0.32 ± 0.01	82.9 ± 5.0	55 ± 2.5

The highest growth rate (0.34 d^–1^) was observed at a 50% leachate concentration; however, productivity dropped further to 132.60 g/m^3^/d, and TN removal efficiency declined to 77%. This pattern suggests that despite active cellular metabolism, stress from elevated pollutant levels limited overall biomass accumulation. Moreover, algae growth was affected by light irradiation, as reported in the literature, and a light intensity of 5000–10000 Lux is typically provided (5000 Lux in the present study).^31^ Although the algae productivity increases with light intensity, high artificial light intensity up to 106250 Lux has resulted in a reduction in NH_4_–N removal (70% decrease). At 100% leachate concentration, productivity (82.90 g/m^3^/d) and TN removal (55%) decreased sharply, confirming that high pollutant and ammonia levels inhibit algal photosynthesis and nutrient uptake. In contrast, the control culture exhibited a growth rate of 0.32 d^–1^ and a productivity of 114 g/m^3^/d, with 70% TN removal, demonstrating that properly diluted and pretreated leachate can perform even better than ideal growth media. Thus, the MD-C-A system is most effective at 10–20% dilution, where nutrient availability, pH balance, and reduced toxicity synergistically promote optimal algal growth and nitrogen removal.

### 3.6 MD-C-ASBR Experiment

#### 3.6.1 Biomass, DO, and pH Profiles in Batch and Continuous Mode MD-C-ASBR System

Figure 4a depicts the variation in biomass concentration (mg/L) over 21 days during seven operational cycles of an Algal Sequencing Batch Reactor (ASBR) and a continuous ASBR (ASBR-Cont.) system. The trends highlight the dynamic behavior of microbial biomass during the fill, react, settle, and decant phases in each cycle and reveal key differences in system stability and biomass retention between batch and continuous modes. An initial decline in biomass concentration was observed during the first three cycles, with values dropping from approximately 580 mg/L to around 200 mg/L in the ASBR and 250 mg/L in the ASBR (Cont.). This decrease reflects the adaptation phase of microbial communities to leachate characteristics and substrate composition, where endogenous respiration exceeds biomass generation due to limited acclimatization and substrate utilization. However, after cycle 3, both systems showed a progressive increase in biomass concentration, indicating microbial adaptation, improved substrate biodegradability, and establishment of a steady state. From cycle 4 onward, the ASBR (Cont.) consistently maintained slightly higher biomass concentrations than the batch ASBR, reaching up to 600 mg/L by day 15. This indicates improved sludge retention, reduced washout, and continuous substrate availability, all of which support stable microbial activity. In contrast, the batch ASBR displayed more pronounced fluctuations, attributed to periodic feeding and settling phases, which temporarily reduced substrate availability and biomass density. Compared to the preliminary study, where the final biomass reached 2100 mg/L, the final biomass in the SBR reactor could reach 620 mg/L due to a shorter HRT of 3 days, required for better real-time control of leachate treatment. An algal SBR used for municipal wastewater treatment achieved algal biomass growth of 3000 mg/L after 22 days without external carbon addition.^33^ However, algal growth was limited by DO limitations, which counteracted the high oxygen demand caused by organic pollutants in the wastewater. Moving to cycle 6–7, both systems achieved near-steady-state conditions, with biomass concentrations stabilizing around 580–620 mg/L. This suggests that the microbial community had fully acclimatized and that the systems had reached dynamic equilibrium between growth and decay. The results demonstrate that while both systems are effective for biomass growth and maintenance, the continuous ASBR provides superior biomass stability and recovery rate due to consistent nutrient supply and lower stress cycles, making it more suitable for long-term leachate or high-strength wastewater treatment applications.

**Figure 4:**
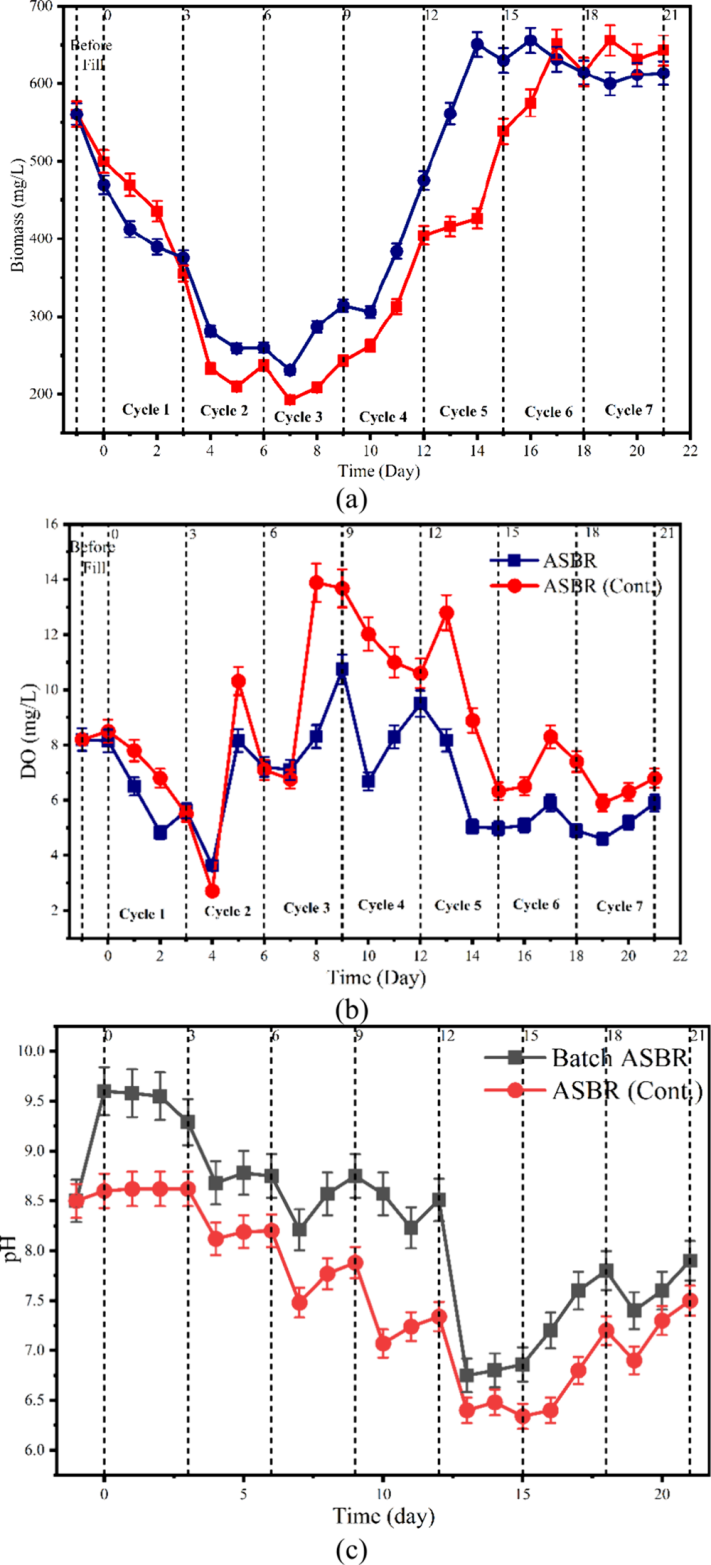
(a) Biomass profile in MD-C-ASBR, (b) DO profile in MD-C-ASBR, and (c) pH profile in MD-C-ASBR system.

Figure 4b illustrates the variation in dissolved oxygen (DO) concentration over a 21-day operational period during seven cycles in both a batch ASBR and a continuous ASBR (ASBR-Cont.) system. DO dynamics reflect the interplay between microbial activity, substrate degradation, and oxygen transfer efficiency, providing valuable insights into the aerobic-anoxic transitions that occur during the treatment cycles. During the initial cycles (Cycle 1–3), both systems exhibited noticeable DO fluctuations. The DO levels in the ASBR ranged from 2 to 10 mg/L, while the ASBR (Cont.) reached higher peaks of 12 to 14 mg/L, suggesting more effective oxygen transfer and reduced oxygen depletion due to continuous feeding and improved mixing. The initial drop in DO after filling reflects high substrate availability and rapid microbial respiration, resulting in oxygen consumption. Every so often, the DO level during the dark phase in an algal photobioreactor study can drop to 0.7–2 mg/L.^31^ The lower DO observed was due to the consumption by aerobic microbes for COD degradation and algal respiration during the night. The calculated algal oxygen production rate and microbial consumption rate in the algal photobioreactor were found to be 81.77 and 60.6 mg/L/d. As the cycles progressed, DO levels increased during the react phase as organic substrate concentration declined, and respiration slowed, allowing oxygen accumulation. It was observed that between cycles 4–6, the ASBR (Cont.) consistently maintained higher DO levels (10–14 mg/L) than the batch ASBR (6–10 mg/L). This behavior indicates enhanced oxygen distribution and stable aerobic conditions in the continuous system, which promotes efficient organic degradation and nitrification. Conversely, the lower DO in the batch ASBR suggests temporary oxygen depletion due to limited mixing and higher oxygen demand during peak microbial activity, resulting in periodic transitions between aerobic and anoxic states. Most algal-alone treatments have not focused on DO measurement during the SBR treatments. However, measuring DO and pH is important for evaluating the efficacy of the algal-bacterial SBR process used for COD and nutrient removal. The DO measurement during an algal-bacterial SBR revealed that algal photosynthesis elevated DO, which was consumed by heterotrophic bacteria for COD removal.^34^ The DO level in the study increased initially and reached 8–10 mg/L after 10 days. Similarly, bacterial biomass produced CO_2_, which was utilized by the algae for their metabolism, thereby establishing symbiosis. Moving to cycle 7, both systems stabilized, maintaining DO concentrations between 4 and 8 mg/L, indicating the establishment of steady-state conditions in which oxygen consumption matched supply. The ASBR (Cont.) outperformed batch mode by sustaining higher, more stable DO levels, which are crucial for maintaining aerobic microbial populations, improving nitrification efficiency, and preventing process inhibition. The results clearly indicate that continuous operation enhances system oxygen dynamics and overall biological treatment performance.

Figure 4c presents the variation in pH over a 21-day operational period encompassing seven cycles in the batch ASBR and the continuous ASBR (ASBR-Cont.) system. The observed pH trends reflect the biochemical transformations occurring in each reactor, including organic degradation, ammonification, nitrification, and CO_2_ generation, which influence the system’s acid–base balance. Initially, both systems exhibited slightly alkaline conditions, with the batch ASBR and ASBR (Cont.) starting at pH values of approximately 9.5 and 8.5, respectively. During the first few cycles (up to 14 days), the pH gradually decreased, indicating the accumulation of acidic intermediates, such as volatile fatty acids (VFAs), produced by the intense microbial oxidation of organic matter. Further, algal photosynthesis releases CO_2_ during the dark phase, and nitrification can decrease the pH of the ASBR. The alkalinity decreased after the 2nd cycle, which explains the pH fluctuations after the seventh day. Interestingly, studies show that some algal species, such as *G. Sulphuraria* can acidify the media during their growth period, resulting in leaching of heavy metals from the solid matrix to the liquid matrix.^32^ In the ASBR, pH fluctuated between 9.8 and 7 across cycles, while in the continuous ASBR, it remained relatively lower (8.5 to 6.5), suggesting better buffering and more stable acid production under continuous feeding. ASBR exhibited sharper pH variations between cycles, particularly around day 12, when it dropped to approximately 6.5, likely due to sudden organic overloading and transient VFA accumulation during the reaction phase. However, recovery occurred in subsequent cycles as VFAs were consumed and alkalinity regenerated, stabilizing pH near 8.0 by the final stage. In contrast, the ASBR (Cont.) maintained a more consistent pH, ranging from slightly acidic to neutral throughout the operation, indicating steady-state conditions and balanced microbial metabolism. Further moving to cycle 7 (day 21), both systems stabilized between pH 7.5 and 8.0, which is optimal for nitrifying and heterotrophic bacteria. The higher pH in ASBR suggests enhanced ammonia stripping, while the lower and stable pH in ASBR (Cont.) favors continuous nitrification. The increase in pH can be correlated with biocarbonate removal, an increase in DO due to algal photosynthesis, endogenous respiration by bacteria and algae (during the night), and symbiosis during algal-bacterial metabolism.^18^ However, increases beyond pH 11 can have a detrimental impact on algal growth and contribute to air pollution through ammonia volatilization. Moreover, Lu et al. reported that a neutral pH of 6 yielded a maximum algal biomass of 3.25 g/L, compared to 1.17 g/L at pH 10.^30^ The study confirms that a higher pH resulted in the conversion of ammonium ions to free ammonia, increasing toxicity and inhibiting algal growth and nitrogen recovery, which was maximum (40.30%) at pH 6. The results confirm that while the batch ASBR undergoes more pronounced fluctuations, the continuous system maintains better pH stability, ensuring sustained microbial activity and improved process resilience for long-term leachate treatment.

#### 3.6.2 Alkalinity Removal in Batch and Continuous Mode MD-C-ASBR System

The comparative performance of M-C-A-SBR and M (Cont.)-C-A-SBR systems over 15 days is shown in Figure 5a, revealing distinct variations in removal efficiency and alkalinity trends, highlighting the influence of operational mode on system stability and ammonia removal. The initial alkalinity at the start of treatment was recorded as 1508 mg/L. During the first cycle, the MD-C-ASBR system exhibited superior alkalinity removal efficiency, achieving 45% removal within 3 days, corresponding to a maximum removal rate of 262 g/m^3^/d. This high initial performance can be attributed to sufficient alkalinity, which supports autotrophic microbial metabolism and promotes stable biochemical reactions during the early treatment phase. However, as the treatment progressed, a gradual decline in alkalinity removal efficiency was observed, driven by alkalinity depletion and reduced microbial activity. In the subsequent second and third cycles, the removal efficiencies dropped significantly to 9.4 and 7.7%, respectively, reflecting the exhaustion of buffering capacity and the inhibitory impact of accumulated nitrogenous intermediates. Interestingly, in later stages (4th and 5th cycles), the system showed recovery in performance, achieving 27 and 33% removal, respectively, likely due to the re-establishment of microbial adaptation and enhanced algal assimilation. In the MD-C-ASBR (Cont.) system, the overall alkalinity removal trend followed a similar pattern; however, the removal rates were relatively higher than those in the batch-operated MD-C-ASBR. This improvement in the continuous mode can be ascribed to the consistent feed supply, which stabilized the alkalinity consumption and maintained microbial activity throughout the process. The alkalinity removal rate during the first cycle of the continuous system was approximately 168 g/m^3^/d, which later ranged between 16 and 50 g/m^3^/d. At the end of the 15-day treatment period, a final alkalinity of 300 mg/L was recorded in both systems, indicating a substantial reduction from the initial value. Furthermore, COD removal showed noticeable improvement by the fifth cycle, suggesting that carbon oxidation and alkalinity utilization were closely coupled processes. Under mixotrophic conditions, efficient TOC removal of 64.27 and 99% of inorganic carbon removal was observed from landfill leachate.^35^ Algal growth in this study was enhanced by carbon assimilation, leading to the formation of 3-phosphoglyceric acid, which reduced the toxic effects of ammonia on algal metabolism. The HCO_3_^–^ concentration in the algal reactor increases at alkaline pH, allowing for the conversion of HCO_3_^–^ into CO_2_, which is subsequently absorbed through the Calvin cycle.^36^ Thus, algae can fix CO_2_ with an efficiency 10–50 times higher than that of plant species. Overall, M-C-A-SBR and M (Cont.)-C-A-SBR systems demonstrated effective alkalinity removal; the MD-C-ASBR (Cont.) exhibited slightly better stability and efficiency, emphasizing the importance of continuous operation in sustaining alkalinity balance and enhancing overall treatment performance. The results indicate that while M-C-A-SBR and M­(Cont.)-C-A-SBR configurations initiate effectively, maintaining alkalinity is vital for sustained removal. The continuous system offers greater stability, whereas the batch mode exhibits higher but less stable removal peaks. The findings underline the need for external alkalinity supplementation or optimized pH control to sustain efficient ammonia removal over extended operational periods.

**Figure 5:**
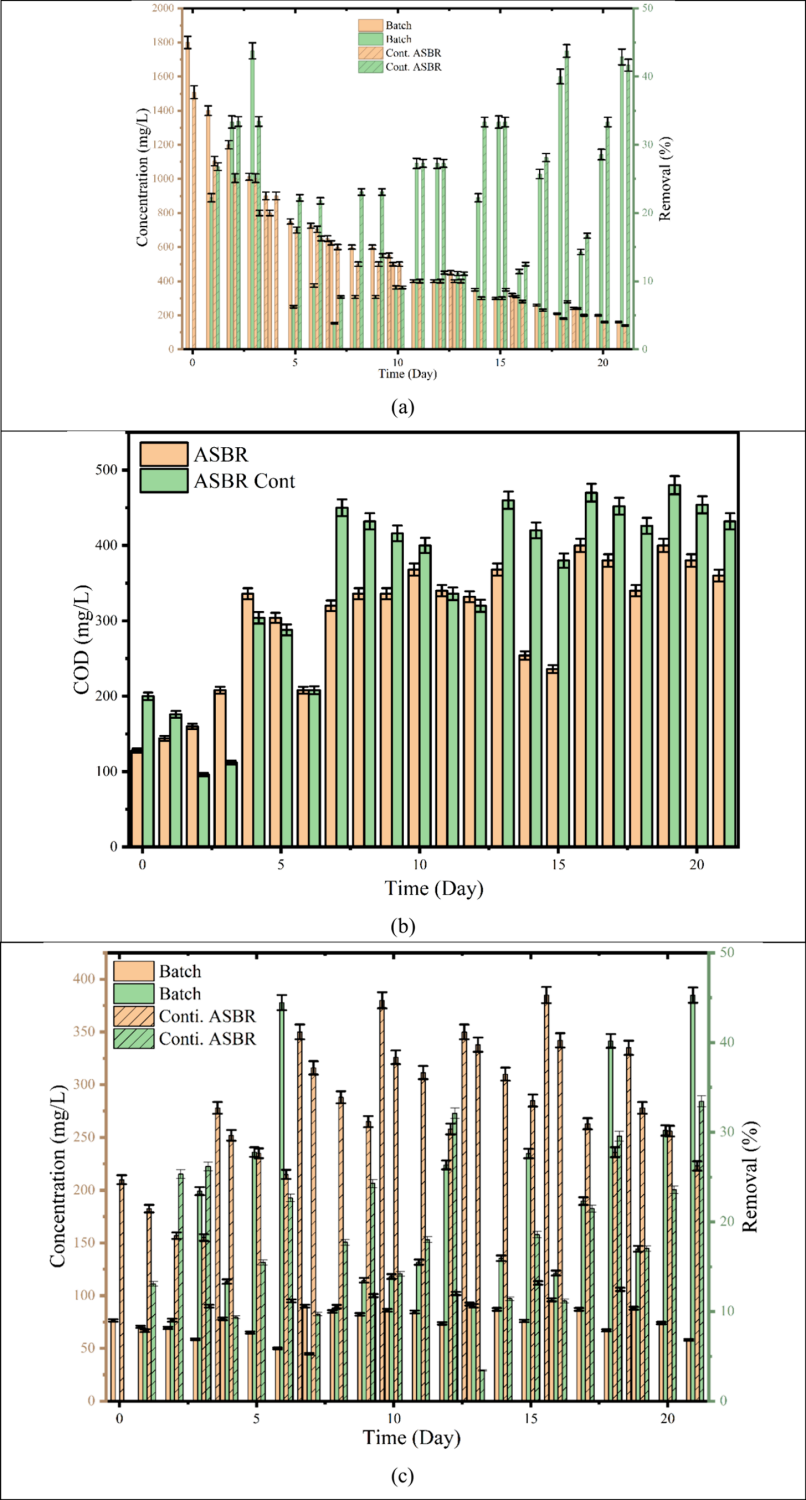
(a) Alkalinity removal in the MD-C-ASBR system, (b) COD removal in the MD-C-ASBR system, and (c) TN removal in the batch and continuous flow ASBR system.

#### 3.6.3 COD Removal in Batch and Continuous Mode MD-C-ASBR System

The variation of COD concentration during treatment using the M-D-C-ASBR and M (Cont.)-D-C-ASBR systems over 15 days demonstrates the comparative degradation efficiency of batch and continuous operational modes (Figure 5b). Initially, the COD concentration was about 200–250 mg/L for both systems. During the early phase (day 1–3A), a noticeable reduction in COD was observed in the M-D-C-ASBR system, reaching approximately 150 mg/L by day 3A, while the M (Cont.)-D-C-ASBR system showed a slightly higher COD of around 200 mg/L, indicating slower degradation under continuous operation. By the 3B phase, the COD levels rose to 300 mg/L in the M-D-C-ASBR and about 350 mg/L in the M (Cont.)-D-C-ASBR, likely due to substrate accumulation or limited microbial adaptation. Furthermore, between days 4 and 6A, both systems exhibited gradual improvement in COD removal efficiency, maintaining COD values between 250 and 300 mg/L in M-D-C-ASBR and 300–350 mg/L in M (Cont.)-D-C-ASBR, reflecting the gradual stabilization of microbial communities. However, during cycle 6B, COD spiked to nearly 500 mg/L in the continuous system, compared to about 400 mg/L in the batch mode, suggesting possible overloading or an imbalance between the substrate feed and microbial degradation rate. From day 7 to day 10, systems reached a semisteady state, with COD values stabilizing around 300–400 mg/L in MD-C-A-SBR and 400–450 mg/L in M (Cont.)­D-C-ASBR, implying sustained microbial activity but limited by substrate recirculation efficiency. Toward the final stages (days 12A-15), both systems showed slight improvement in organic matter degradation, with COD concentrations stabilizing around 350–400 mg/L for MD-C-A-SBR and 400–450 mg/L for the continuous system. The higher COD retention in the M (Cont.) D-C-ASBR system suggests that continuous inflow caused incomplete degradation due to reduced retention time, while the batch system benefited from cyclic aeration and sufficient contact time for microbial oxidation. The algae can remove COD from landfill leachate in mixotrophic and heterotrophic conditions when an algal-bacterial consortium is used in the photobioreactor. The landfill leachate treatment using an algal-bacterial mixed culture shows promising COD removal of 77.14–81.0% (removal rate of 40.10–42 mg/L/d) in the first cycle of the SBR, and efficient removal was achieved until the 11th day.^37^ However, COD removal decreased after day 11 due to the exhaustion of biodegradable compounds and the toxicity of inorganic phenolic compounds. The MD-C-ASBR exhibited better organic removal efficiency throughout the study period due to improved oxygen transfer, cyclic operation, and enhanced microbial acclimatization. In contrast, although the M (Cont.)­D-C-ASBR showed higher removal rates initially, but it experienced fluctuation and accumulation of organic load during continuous operation, indicating the need for optimized flow rate and aeration balance for sustained COD removal efficiency.

#### 3.6.4 TN Removal in Batch and Continuous Mode MD-C-ASBR System

The variations in total nitrogen (TN) removal efficiency and TN concentration for the MD-C-A-SBR and M (Cont.)­D-C-ASBR systems, when operated for 15 days, exhibit distinct operational behaviors and nitrogen transformation patterns in batch and continuous modes (Figure 5c). At the start (day 0), both systems exhibited low TN removal (<5%), corresponding to TN concentrations of approximately 1800 mg/L, as the microbial and algal communities were in the acclimation phase. By day 2, the MD-C-ASBR system achieved around 10% TN removal, while the M (Cont.)­The D-C-ASBR system showed a slightly higher efficiency of 18–20%, likely due to the continuous nutrient supply and more stable microbial-nutrient interactions. On day three, M-D-C-A-SBR and M (Cont.) D-C-ASBR systems displayed similar removal rates of approximately 25%, reflecting enhanced nitrification-denitrification and algal uptake activity. A significant improvement was observed on days 5 and 6 in the MD-C-ASBR system, where removal peaked at nearly 45%, while the M (Cont.)­D-C-ASBR system maintained a moderate efficiency of 25–30%. The treatment of municipal wastewater was carried out with microalgae, *Scenedesmus sp.,* at an HRT of 4–10 days, and the treatment achieved higher TN removal of 81% at HRT of 4 days compared to 54.8% at 6 days.^29^ However, longer HRT resulted in a lower nutrient load into the reactor, promoting a larger cyanobacterial population than green algae. Moreover, when HRT was extended to 10 days, the algal system reflected a higher *Scenedesmus sp.* population and limiting nitrogen conditions in the reactor. The N:P ratio during treatment was 7 at an HRT of 10 days and 34 at an HRT of 6 days. However, the optimum N:P ratio of 16 was maintained when the reactor HRT was set to 4 days, resulting in the highest TN removal and optimal algal growth. A maximum TN removal rate of 10 mg/L/d was observed at an HRT of 4 days, compared to 3.25 mg/L/d at an HRT of 10 days, due to a higher volumetric loading rate. In the present study, the N:P ratio increased from 1.7 (day 0) to 6.1 (day 21) in the batch SBR reactor, whereas it increased from 4.5 (day 0) to 21 (day 21) in the continuous SBR reactor. The optimal N:P ratio is considered to be 16 (Redfield ratio), which leads to efficient nutrient removal and algal growth in an algal photobioreactor.^38^ This indicates that nutrient assimilation in the continuous reactor was more effective due to a higher TN loading rate and gradually improved during successive cycles, thanks to efficient TP removal in each cycle.

The sudden increase in the batch system indicates an optimal balance between aeration, organic load, and microbial activity, promoting effective nitrogen conversion. However, after day 7, TN removal in both systems showed a fluctuating pattern: the MD-C-ASBR removal ranged from 10 to 20%, whereas the M (Cont.)-D-C-ASBR system varied between 15 and 25%, with slight peaks on days 9 and 12. The TN concentration profile (right axis) showed that the M (Cont.)­D-C-ASBR maintained higher TN levels (400–600 mg/L) compared to MD-C-ASBR (below 300 mg/L), indicating more consistent nitrogen retention under continuous inflow conditions. From days 10 to 15, M-D-C-ASBR and M (Cont.)-D-C-ASBR systems demonstrated stabilization in TN profiles, with M-D-C-ASBR showing minor oscillations around 150–250 mg/L and M (Cont.)­D-C-ASBR around 400–500 mg/L. Previous research has focused on commercially available algal species for landfill leachate treatment; however, algal species collected from the natural environment are more resilient to shock load.^35^ Furthermore, a mixed microalgal culture consisting predominantly of Chlorella sp. was used at a controlled N:P ratio of 14 for landfill leachate, achieving 88.64% TN and 98.81% NH_4_–N after 7 days. It was shown that phosphorus addition to maintain the N:P ratio improved the TN removal efficiency from 69.3% (without P addition) to 88.6% (With P addition). However, no phosphorus was added in the present study, and the N:P ratio increased to 21 after 7 cycles in the ASBR (cont.), which could lead to phosphorus limitation if no external phosphorus is added. These results suggest that the batch-operated system provided better nitrogen removal efficiency, especially during peak cycles, due to controlled aeration, sufficient retention time, and effective alternation between aerobic and anoxic conditions, which are favorable for nitrification and denitrification. In contrast, the continuous system offered more stable but lower removal efficiency, likely due to reduced contact time and dilution effects. The MD-C-ASBR exhibited superior TN removal performance, whereas the M (Cont.) D-C-ASBR maintained better operational stability, but at the cost of slower nitrogen conversion. Xiao et al. have reported enhancement in TN removal (up 88%) and energy saving due to extended operational cycle (21–33 days) in a membrane bioreactor for landfill leachate treatment.^39^ Further, the extended time reduced fouling from 0.42 to 0.25 kPa/d and reduced operational cost.

The rates of removal of alkalinity, total nitrogen (TN), and total phosphorus (TP) in the MD-C-ASBR and MD-C-ASBR (Cont.) systems varied considerably across cycles, indicating distinct system dynamics under batch and continuous operation (Table 4). In the MD-C-ASBR system, the highest alkalinity removal rate of 262 g/m^3^/d was achieved during the first cycle, corresponding to strong microbial activity and sufficient buffering capacity. Subsequent cycles showed a gradual decline, stabilizing between 16 and 50 g/m^3^/d from the third to seventh cycles. Conversely, the continuous system exhibited a lower initial removal rate (168 g/m^3^/d) but maintained more consistent performance (33–65 g/m^3^/d), suggesting better stability. TN removal followed a similar trend; MD-C-ASBR showed fluctuating rates ranging from 4.26 to 13.33 g/m^3^/d, while the continuous system maintained higher, steadier rates of 18.28–29 g/m^3^/d, reflecting improved nitrification and denitrification efficiency under continuous substrate supply. Similar to this study, ozonation as a pretreatment has been combined with microalgae to achieve an ammonia removal rate of 25.85 mg/L/d (ammonia removal of 81.60%).^40^ On the other hand, a comparatively lower ammonia removal rate of 3.8–14.5 mg/L/d was achieved in a bubble column photobioreactor for landfill leachate treatment using an algal-bacterial consortium at a dilution of 10%, which reduced the nitrogen loading in the reactor.^37^ Furthermore, dilution requires more water resources, resulting in a negative life cycle impact of algal treatment. In this study, the dilution was limited to 50% to ensure proper nutrient loading in the reactor and maintain the N:P ratio. The batch system demonstrated higher peak efficiencies, but with greater variability, while the continuous system exhibited steady, yet moderate, nutrient removal. This suggests that cyclic aeration in batch mode enhances microbial metabolism and nutrient conversion, whereas continuous mode provides operational stability and sustained removal performance over extended cycles.

**Table 4:** Rate of Removal of Alkalinity, TN, and TP in the MD-C-ASBR System

	rate of removal (g/m^3^/d)					
	alkalinity		TN		TP	
SBR experiment cycle no.	MD-C-A SBR	MD-C-ASBR (Cont.)	MD-C-A SBR	MD-C-ASBR (Cont.)	MD-C-A SBR	MD-C-ASBR (Cont.)
1	262	168	5.96	18.28	1.33	2.66
2	25	65	13.3	21	1.16	0.33
3	16	50	4.26	3.33	1.83	0.33
4	50	50	8.78	23.5	0.67	0.83
5	50	50	5	21.6	0.33	0.33
6	46	46	9.67	22.33	0.50	0.76
7	40	33	13.33	29	0.76	1

#### 3.6.5 TP Removal in Batch and Continuous Algal SBR

The variation in total phosphorus (TP) removal efficiency for ASBR-Batch and ASBR-Cont. Systems over a 21-day operational period demonstrate significant differences in performance and adaptability between the two reactor modes (Figure 6a). During the initial phase (day 3), the ASBR-Batch system achieved 8.70% TP removal, whereas the ASBR-Cont. The system demonstrated superior performance with a 17.50% removal rate, attributed to its continuous nutrient flow, which enabled stable phosphorus uptake by microorganisms. Moving to day 6, the ASBR-Batch system recorded a moderate increase to 13.4%, while TP removal in the ASBR-Cont., declined sharply to around 4.70%, possibly due to nutrient shock or microbial imbalance under constant feed conditions. A remarkable peak in TP removal was observed in the ASBR-Batch system on day 9, where efficiency reached 31.60%, the highest among all cycles, while ASBR-Cont. showed minimal improvement at 5.50%. This substantial increase in the batch mode can be linked to the cyclic aeration pattern and the alternating anaerobic–aerobic conditions, which enhance phosphorus release and uptake by polyphosphate-accumulating organisms (PAOs). In optimum conditions, such as continuous light, excess N and neutral pH, 100% PO_4_–P removal at a faster rate was observed at an initial TP concentration of 15–300 mg/L during leachate treatment due to intense luxury uptake by PAOs.^41^ Most studies have observed that P removal in algae is a faster process than N removal, due to rapid cellular consumption and accumulation in the form of intracellular polyphosphate (poly-P) in the algal cells.^42^ On day 12, both systems showed similar performance with removal efficiencies of 16.8% (Batch) and 17.2% (Cont.), indicating a temporary equilibrium between the two operational modes as microbial communities stabilized. By day 15, TP removal declined uniformly in both systems to 9%, likely due to nutrient exhaustion or reduced PAO activity. In the subsequent cycles (days 18–21), a recovery in phosphorus removal was observed.

**Figure 6:**
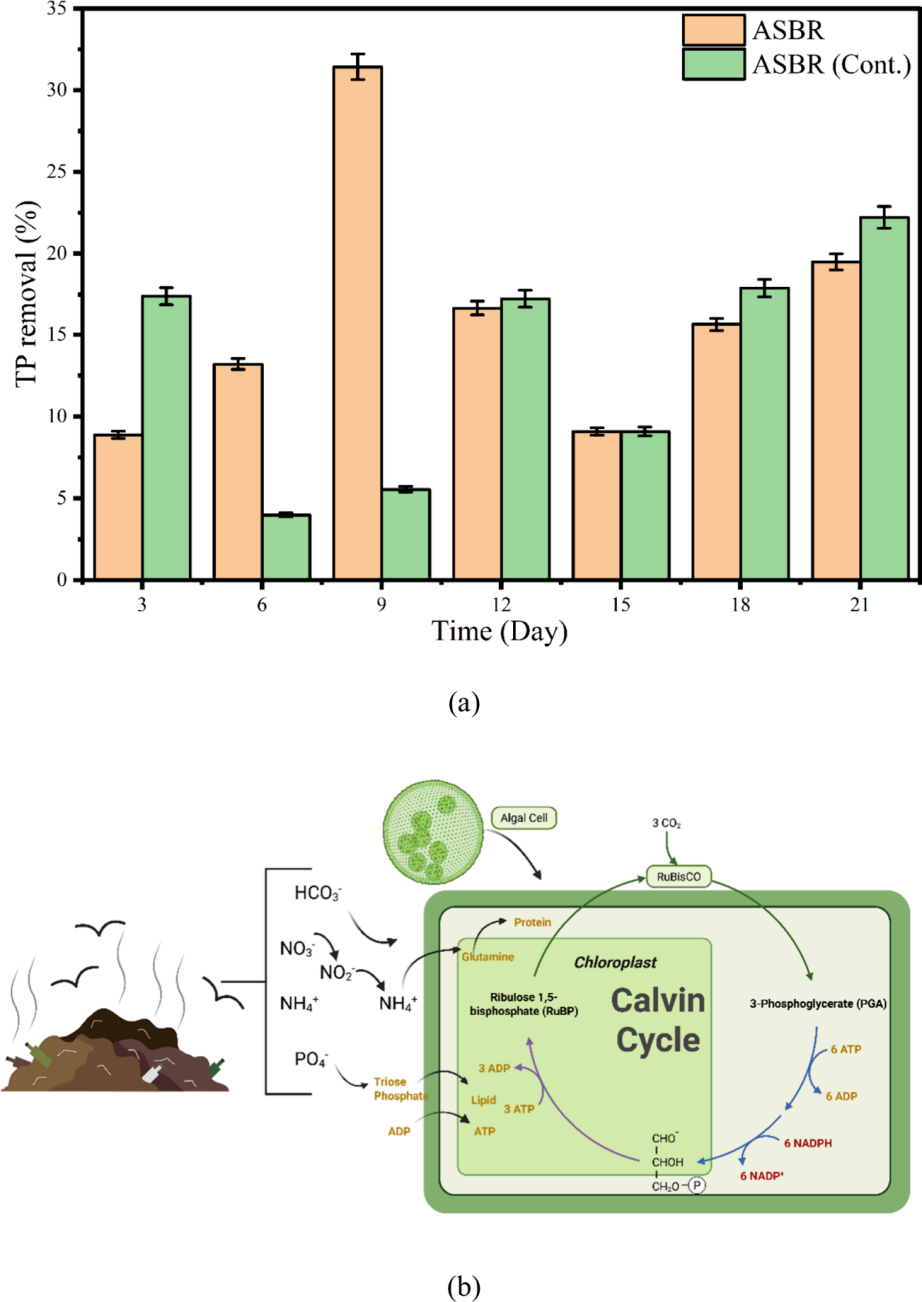
(a) TP removal in the ASBR System and (b) mechanism of P intake into the algal cell.

The ASBR_Batch reached 15.7 and 19.4% on days 18 and 21, respectively, while the ASBR-Cont., system outperformed slightly with 17.80 and 22.40% during the same period. The gradual improvement toward the end of the experiment suggests microbial adaptation and balanced phosphate metabolism. For TP, MD-C-ASBR achieved moderate removal (0.33–1.83 g/m^3^/d), whereas the continuous mode recorded 0.33–2.66 g/m^3^/d, with the highest values observed in early cycles due to active algal uptake and microbial assimilation. The landfill leachate is considered P-limited, which can be a bottleneck during the continuous operation of the algal reactor and sometimes requires external phosphorus (P) to balance the N:P ratio. Though intracellular P assimilation increases with P loading, the TP removal and algal growth can decrease due to an alteration in the N:P ratio at high P loading.^27^ For instance, algal biomass was highest at an N:P ratio of 16 (28 g/m^2^); however, when more P was added to change the N:P ratio to 3, the biomass reduced to 25.60 g/m^2^.^40^ The assimilation of P loading onto algal cells occurs through the Calvin cycle, through which NO_3_–N is converted into NH_4_–N and subsequently into glutamine and protein (Figure 6b). Similarly, the P loading is converted into triose phosphate, which can be used to form lipids in algal cells. The poly-P stored in the algal cell can be visualized by fluorescence microscopy to confirm algal growth, as reported in our earlier work.^43^ Excess P loading caused toxicity to the algae, and lipid formation was enhanced rather than algal biomass. During wastewater treatment using an algal bioreactor, predominantly containing Cyanobacteria, the TP removal rate was observed to be 1.02–3.06 mg/L/d at HRT of 3–10 days, with corresponding removal efficiencies of 83–23–98%.^29^ The ASBR-Batch system exhibited higher peak efficiency but greater fluctuations in TP removal, driven by periodic aeration and feed cycles promoting biological phosphorus uptake. In contrast, the ASBR-Cont., system demonstrated more consistent, though lower, removal during early stages, with improved stability and efficiency in later cycles due to continuous nutrient exposure. These results show that batch operation offers high-intensity removal potential under optimal conditions, whereas continuous operation ensures long-term stability and uniform phosphorus reduction performance.

#### 3.6.6 Metal Removal

The metal removal percentage against the metal is listed in Figure 7a. In the first cycle, removal efficiencies were relatively low, with Pb at around 15%, Cu at 10%, Mn at 8%, Zn at 90%, and Fe at 95%. This indicates that Zn and Fe were more readily removed due to their higher affinity for adsorption sites. During the second cycle, Pb removal increased to nearly 60%, Cu to 20%, and Mn to 12%, while Zn and Fe remained consistently high, above 90%, indicating early stage stabilization for these two metals. By the third cycle, Pb removal reached approximately 70%, Cu rose sharply to 45%, and Mn improved to 18%, reflecting enhanced biological activity and better interaction between the metal ions and biomass. The fourth and fifth cycles demonstrated substantial improvements: Pb achieved nearly complete removal (95–100%), Cu and Mn reached about 25 and 30%, respectively, and Zn and Fe maintained removal efficiencies of 98–100%. This indicates that with successive cycles, the biofilm matrix and algal-metal complexation sites became more active and effective. In the sixth cycle, Pb and Zn removal remained consistently high at 100%, while Cu removal improved to 35% and Mn reached 40%. Fe stabilized around 96%, suggesting steady-state conditions and full system maturity. The final (seventh) cycle exhibited the highest overall performance, with Pb removal sustained at 100%, Cu reaching 50%, Mn peaking at 55%, Zn remaining at 98%, and Fe being stable at approximately 97%. The removal of heavy metal ions, such as Fe, Zn, Cu, Pb, and Cr, from dumpsite leachate was carried out using an algae and cyanobacteria consortium at an initial metal concentration of 5–10 mg/L and an algal biomass of 0.8–1.6 g/L.^44^ Among these metals, the Pb adsorption was highest with a biosorption capacity of 7.03 mg/g at pH 4–5, followed by Cu, Zn, Fe and Cr. The high Pb adsorption was attributed to the presence of carboxyl and sulfonate groups on the algal biomass cells, which act as ligands that bind Pb. Similarly, *Spirilluna Sp.* has shown its efficiency in removing COD (52%) along with heavy metals such as Fe (93%), Mg (42.4%), and Mn (91.5%) from landfill leachate after 10 days in a batch-fed algal photobioreactor.^45^ Li et al. inferred that the major mechanisms behind heavy metal removal were extracellular adsorption and intracellular uptake and measured the amount of Zn uptake by the algal biomass *Stichococcus bacillaris*, which is resistant to Zn toxicity.^46^ It was observed that Zn uptake decreased within the first 10 h, as biosorption equilibrium was reached at a Zn concentration of 2.3 mg/L in the synthetic sample. The Zn adsorption capacity was observed as 15–19 mg Zn/g algal dry mass at an initial Zn concentration of 2–3 mg/L. In the continuous reactor of the same study, nutrient limitation and inefficient light penetration were observed, which hindered algal growth and Zn biosorption, resulting in around 85% removal after 14 days.^46^ Furthermore, when the algal treatment was carried out with real mine dump leachate, 84% Zn removal was achieved after 4 days. The consistent and near-complete removal of Pb, Zn, and Fe indicates strong metal-binding capacity of the microbial-algal consortium and efficient coprecipitation or ion exchange mechanisms. The gradual increase in Cu and Mn removal efficiencies across cycles suggests the time-dependent development of metal-specific adsorption sites and enzymatic activity. The system demonstrated exceptional adaptability and sustainability in multimetal removal, achieving nearly complete elimination of Pb, Zn, and Fe, while showing progressive improvements in Cu and Mn through enhanced biosorption and biological metal-uptake efficiency.

**Figure 7:**
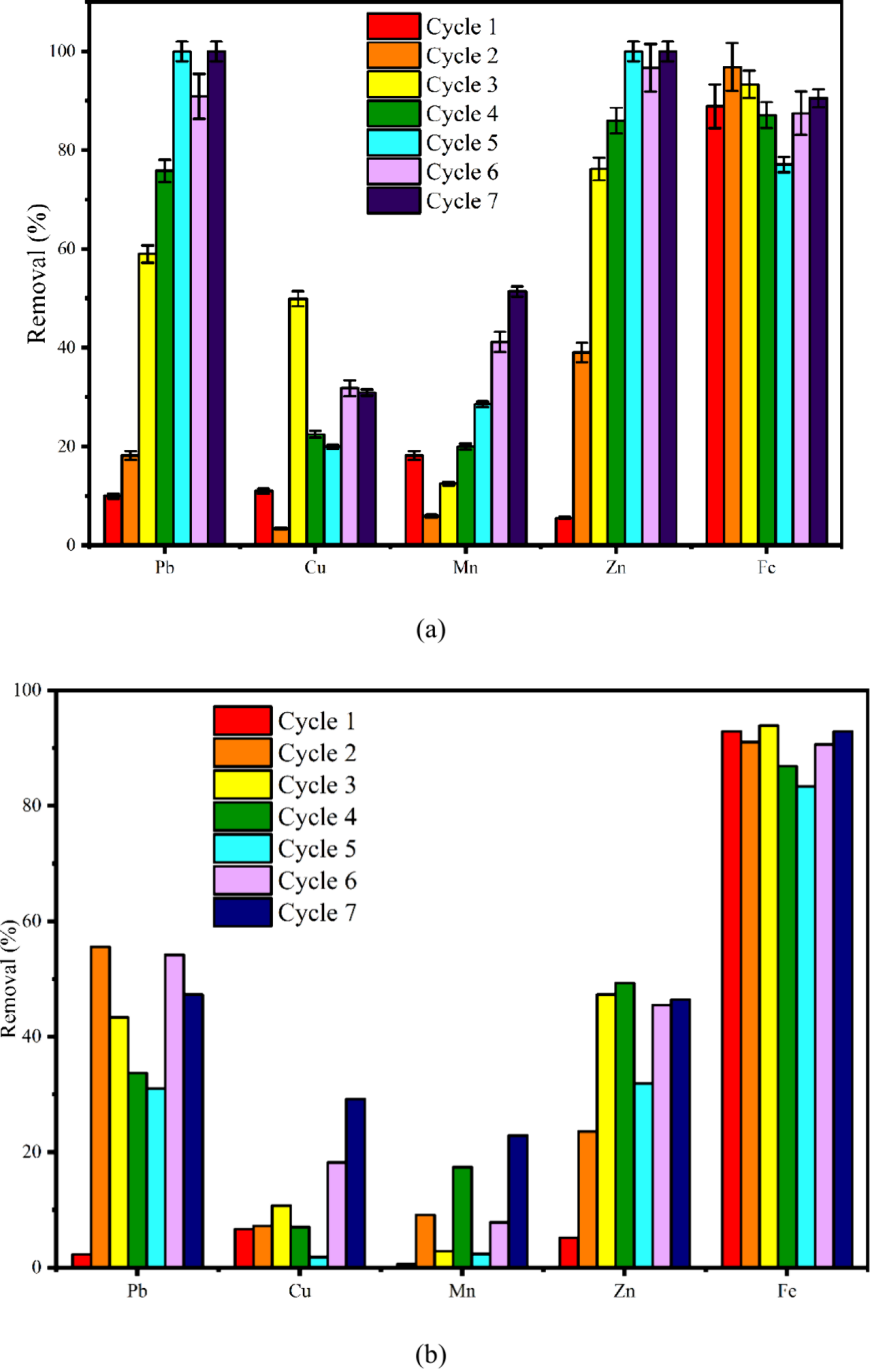
Metal removal in the ASBR reactor (a) batch and (b) continuous.

The removal efficiencies of heavy metals (Pb, Cu, Mn, Zn, and Fe) across seven operational cycles reveal the progressive response and adaptation of the treatment system toward multimetal removal under varying environmental and biological conditions (Figure 7b). In the first cycle, Pb removal was minimal, at around 5%. Cu and Mn were recorded below 10%, while Zn achieved 20%. Fe exhibited the highest removal efficiency, at nearly 95%, indicating a strong adsorption and precipitation potential for iron due to its higher reactivity and ionic affinity. During the second cycle, Pb removal improved significantly to approximately 55%, while Cu remained low at around 10%, Mn at approximately 8%, Zn at 30%, and Fe above 90%, suggesting enhanced microbial and algal uptake activity, especially for Pb and Zn. The third cycle maintained moderate performance, with Pb removal around 45%, Cu and Mn between 10 and 15%, Zn showing the greatest improvement at 55%, and Fe remaining nearly constant at 95%. By the fourth cycle, Pb removal dropped slightly to 33%, possibly due to site saturation or ionic competition. Cu remained near 12%, Mn improved to 16%, Zn sustained around 60%, and Fe stabilized around 85%, reflecting steady-state system conditions. In the fifth cycle, Pb removal declined further to 25%, while Cu and Mn removal increased to 10 and 20%, respectively; Zn decreased slightly to 35%, and Fe remained steady at around 88%. A strong recovery trend was observed during the sixth cycle, with Pb increasing to 55%, Cu reaching 18%, Mn dropping slightly to 8%, Zn maintaining 50%, and Fe around 90%. By the seventh cycle, Pb stabilized at 50%, Cu reached its highest value yet at approximately 30%, Mn improved to 22%, Zn remained constant at around 48%, and Fe maintained 93% removal. The consistent high Fe and Zn removal across all cycles indicates strong chemical and biological affinity for these metals, while the gradual improvement in Pb, Cu, and Mn suggests progressive microbial adaptation, enhanced biofilm formation, and surface complexation. Richards et al. studied the removal of heavy metals from leachate using microalgae and reported that, after 10 days, 95% of the heavy metals were removed using the microalgae *Nanochloropsis gaditana* and *C. muelleri*.^47^

## 4 Biofuel Production from Algal Biomass

The biofuel production potential of the system was evaluated by determining the calorific value of the dried algal biomass, which was found to be 16.50 KJ/kg. This calorific value reflects the energy stored in algal biomass, primarily due to its lipid content, which typically ranges from 20 to 40% depending on species, nutrient availability, and cultivation conditions. Lipids are the primary contributors to the energy density of algal biomass, followed by carbohydrates and proteins.^18^ The obtained calorific value has good agreement with the value reported for Chlorella and Spirulina.^48^ On the other hand, the calorific value of biodiesel was 36.00 KJ/g, respectively.^49^ This indicates that while algal biomass contains a lower energy content per gram, it provides a sustainable and renewable alternative with additional environmental benefits, such as nutrient recovery and CO_2_ sequestration. The detailed energy analysis for a scale-up design required to treat 100 L of leachate is provided in the supplementary section. Moreover, integrating algal treatment for landfill leachate offers dual advantages: simultaneous pollutant removal and biomass generation with energy value. Therefore, the algal biomass produced using landfill leachate could be useful in minimizing the overall cost of treatment.

## 5 Limitations of the Study

Several limitations have been identified that warrant further investigation before implementing the hybrid microwave-coagulation-algal (M-C-A) photobioreactor system on a large scale, despite the promising results obtained. First, the microwave reactor exhibited high energy consumption per unit volume, which could limit its economic feasibility at larger scales. The process efficiency is sensitive to flow rate and temperature variations, and maintaining uniform microwave distribution throughout the reactor volume remains a challenge, potentially leading to localized overheating or uneven treatment. The coagulation stage involves the use of chemical coagulants, such as FeCl_3_, which generate sludge requiring additional handling, disposal, or valorization steps, despite significant improvements in turbidity and COD removal. Furthermore, the algal photobioreactor’s performance was found to be influenced by environmental factors such as light intensity, temperature, and pH, which are difficult to maintain consistently under outdoor or large-scale conditions. Algal growth was also affected at higher leachate concentrations due to ammonia toxicity and nutrient imbalance, suggesting the need for optimized dilution and pretreatment strategies. In addition, phosphorus limitation observed in later treatment cycles restricted nutrient removal efficiency and biomass productivity, highlighting the requirement for nutrient supplementation or coculturing with other microalgal species to maintain stable performance. The continuous flow system requires precise hydraulic control and regular maintenance to prevent clogging, biofilm formation, and flow irregularities. The analytical limitations also limit the capture of real-time variations in metal and nutrient concentrations, which could provide deeper insight into process kinetics. Moreover, the present study did not extensively evaluate long-term operational stability, fouling tendencies, and the potential accumulation of residual contaminants within the system. Furthermore, the economic and environmental assessments, such as energy balances, cost optimization, and life-cycle impacts, were beyond the present scope but are critical for assessing practical viability. Therefore, while the hybrid M-C-A system shows high potential for sustainable leachate treatment, addressing these technical, operational, and economic constraints is essential to ensure its scalability, efficiency, and environmental compatibility in real-world applications.

## 6 Conclusions and Future Scope

The present study successfully demonstrated an integrated hybrid system combining microwave, coagulation, and algal photobioreactor processes for the efficient treatment of landfill leachate in batch and continuous-flow modes. The microwave pretreatment effectively reduced the ammonia concentration by 83.60% at 95 °C, mitigating toxicity and enhancing the biodegradability of the leachate for subsequent biological treatment. Furthermore, the coagulation using ferric chloride achieved substantial removal of turbidity (90%) and COD (76%), complementing the microwave stage. The algal photobioreactor demonstrated remarkable nutrient and heavy metal removal performance, achieving a total nitrogen (TN) removal efficiency of 77% at a 50% leachate dilution and a TN removal rate of 23.50 g/m^3^/d under continuous operation. Likewise, the total phosphorus (TP) removal rate reached 2.66 g/m^3^/d, while Zn^2+^ and Pb^2+^ were completely removed, and Fe removal exceeded 90%. The algal biomass produced had a calorific value of 16.50 MJ/kg, indicating its potential for biofuel generation. Overall, the continuous hybrid M-C-A system proved more efficient than batch operations, offering improved scalability, operational stability, and sustainability. The synergy between physicochemical and biological mechanisms significantly enhanced treatment efficiency, underscoring the potential of this hybrid system as a feasible, eco-friendly solution for landfill leachate management and resource recovery.

Future research should focus on scaling up the hybrid M-C-A system to a pilot or industrial level to evaluate its performance under real-time operational and environmental conditions. Optimization of microwave power, irradiation time, and flow rate can further improve energy efficiency while maintaining high pollutant removal. Ammonia emitted during microwave heating can be captured and used as green ammonia for energy storage and hydrogen conversion. Additionally, integrating real-time monitoring and automation systems would help regulate operational parameters, such as temperature, pH, and nutrient load, to enhance reactor stability and consistency in performance. Furthermore, exploring alternative coagulants or natural alternatives, such as biobased flocculants, could reduce chemical use and environmental impact. In the algal stage, strain selection and genetic enhancement of native microalgae could improve tolerance to high pollutant loads and enhance lipid accumulation for biofuel production. Combining the algal system with downstream processes such as anaerobic digestion and biodiesel conversion can contribute to circular bioeconomy goals by utilizing the generated biomass. Additionally, life cycle assessment (LCA) and techno-economic analysis are also crucial for evaluating the system’s energy balance, cost-effectiveness, and carbon footprint. Advanced mathematical models are required to understand the synergies between these processes, and machine learning and artificial intelligence tools can be integrated into the experimental procedure to reveal mechanisms and enhance our understanding of these synergies. Furthermore, studies should investigate the coupling of this hybrid technology with other advanced oxidation processes or electrochemical methods for treating highly recalcitrant compounds. Overall, the hybrid microwave-coagulation-algal (M-C-A) system holds significant promise as a next-generation, sustainable treatment approach for complex wastewaters, such as landfill leachate, offering dual benefits of pollution mitigation and resource recovery. The continued research and engineering innovation can facilitate its transition from laboratory-scale demonstration to full-scale application for smart, energy-efficient wastewater management.

## Supplementary Material



## Data Availability

The data underlying this study are not publicly available due to confidentiality and institutional restrictions. However, the data are available from the corresponding author upon reasonable request for research purposes.

## Notes

Table S1. Elemental composition of dried sludge from the CF experiment with EDAX; Fig. S1. (a) EDAX and (b) SEM analysis of dried sludge from the CF experiment; Fig. S2. (a) final concentration of COD and ammonia, and (b) pH profile in different units during the batch coupled MW-C-algal process; and scale-up design (PDF)
