# Supplementary material for: Treatment of Landfill Leachate Using an Advanced Microwave Reactor Coupled with a Batch and Continuous Algal Photobioreactor
Source: ACS Omega. 2026 Mar 18;11(12):19547–66. doi: 10.1021/acsomega.5c13308 (PMC13044591; doi:10.1021/acsomega.5c13308)
Supplement: Supplementary file 1 [file ao5c13308_si_001.pdf]

Supplementary file

Treatment of landfill leachate using an advanced microwave reactor coupled with a batch and continuous algal photo-bioreactor

Binay Kumar Tripathy[1], Ranjeet Kumar Mishra*[1], Mathava Kumar[2]*,

[1] Manipal Institute of Technology, Manipal Academy of Higher Education, Manipal, India.

[2]Environmenal and Water Resources Engineering Division, Department of Civil Engineering, Indian Institute of Technology Madras, Chennai, Tamil Nadu, India.

*Corresponding author details:

mathav@iitm.ac.in (Mathava Kumar), ranjeet.mishra@manipal.edu (Ranjeet Kumar Mishra)

Table S1. Elemental Composition of dried sludge from CF experiment with EDAX

Element	***Weight%***	***Atomic weight%***
Carbon	28.88	48.07
Oxygen	18.32	22.90
Sodium	03.16	02.75
Magnesium	01.60	01.31
Aluminum	01.09	00.81
Silicon	01.03	00.73
Sulfur	07.05	04.39
Chloride	14.41	08.13
Potassium	13.73	07.02
Calcium	00.25	00.12
Iron	10.49	03.75

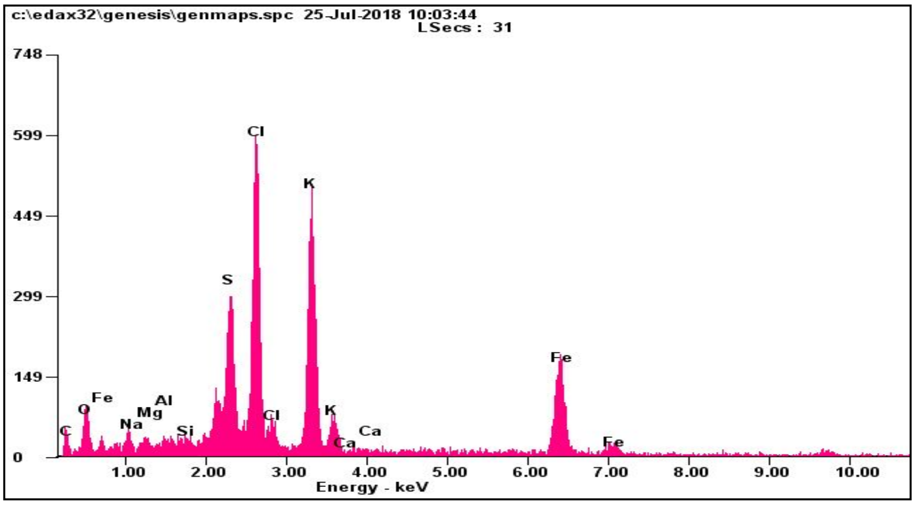

(a)

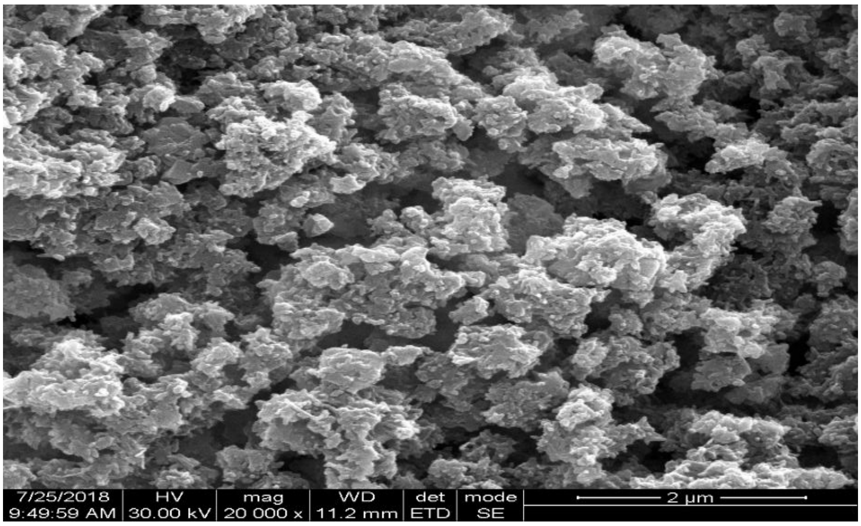

(b)

Fig. S1. (a) EDAX and (b) SEM analysis of dried sludge from CF experiment

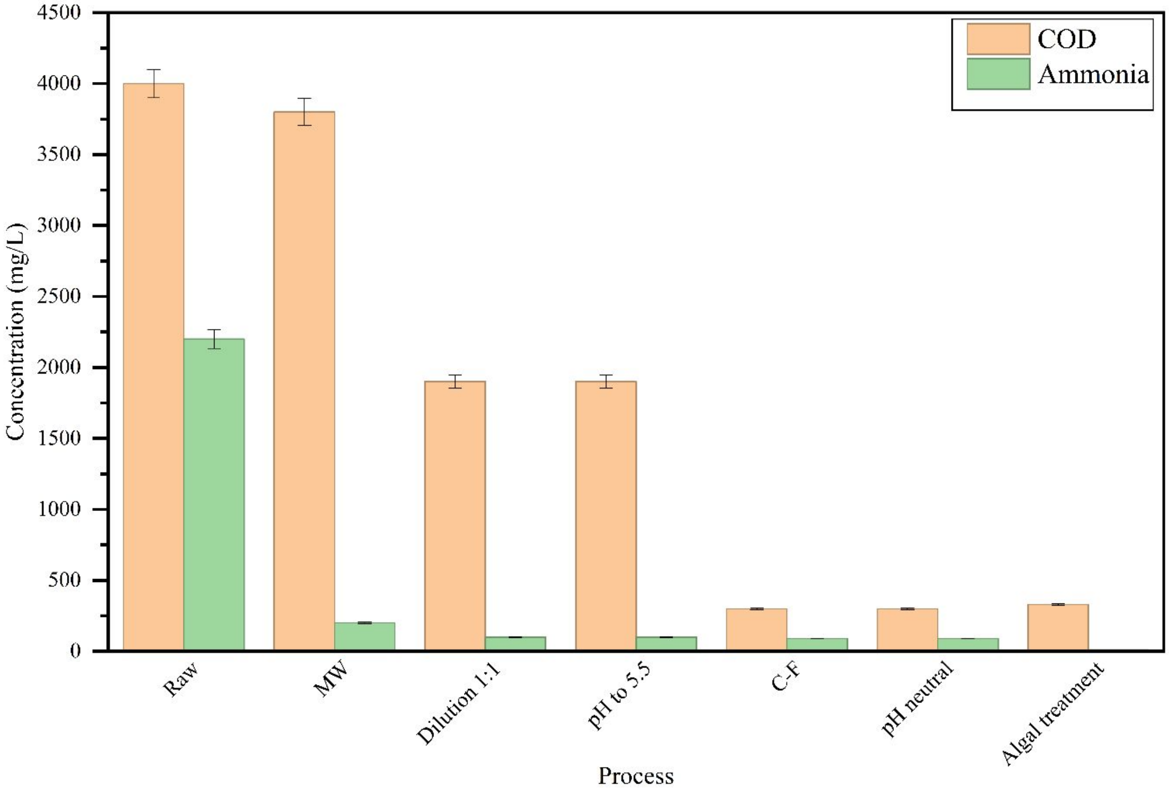
COD
Ammonia
Concentration (mg/L)
4500
4000
3500
3000
2500
2000
1500
1000
500
0
Raw
MW
Dilution 1:1
pH to 5.5
C-F
pH neutral
Algal treatment
Process

(a)

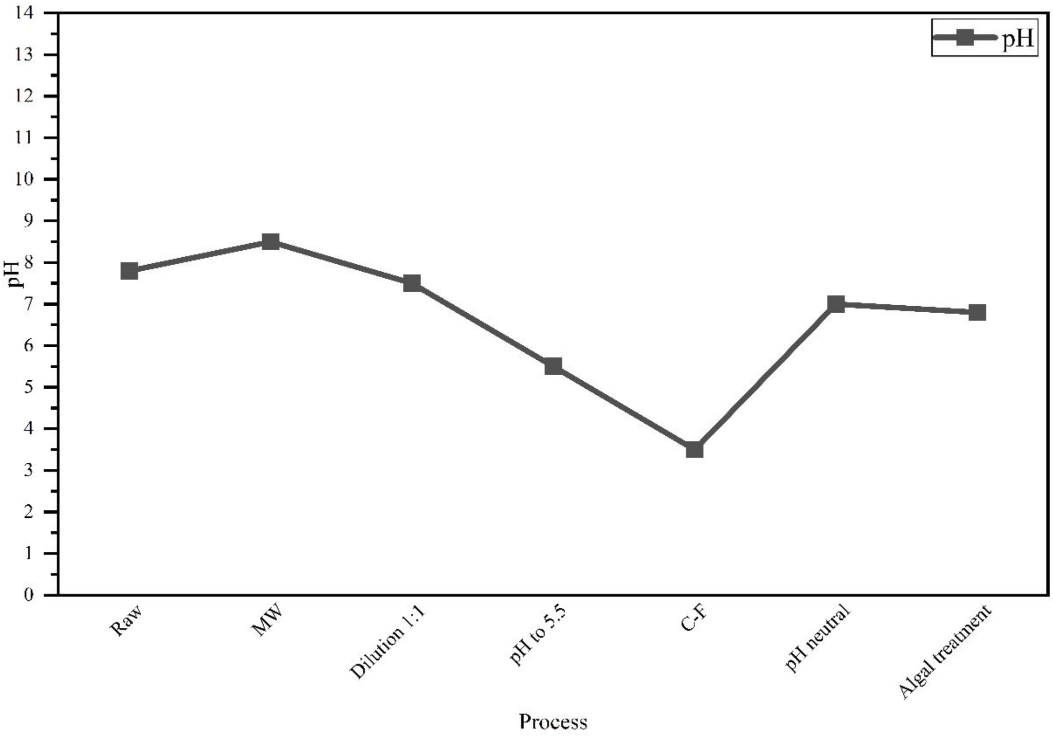
pH
14
13
12
11
10
9
8
7
6
5
4
3
2
1
0
Raw
MW
Dilution 1:1
pH to 5.5
C-F
pH neutral
Algal treatment
Process

(b)

Fig. S2. (a) Final concentration of COD and ammonia, and (b) pH profile in different units during the batch coupled MW-C-algal process

Scale-up design

A scale-up design for the treatment of 100 L of leachate using the MW system was carried out. The MW energy required to treat 100 L of leachate, considering a flow rate of 0.6 Lh-1, was calculated (Eq. (S1)).

$$MW\ Energy = power \times \frac{volume}{flow} \qquad (S1)$$

$$= 500W \times \frac{100\ L}{1000\,\frac{Wh}{KWh} \times 0.6\ Lh^{-1}} = 83\ kWh = 3600KJ$$

Energy required in CF process for 100 L of leachate would be 0.54 KWh (Table 5.9) and energy required for lighting in algal reactor would be 0.432 KWh (considering 18W bulb for 24 h) in laboratory. However, during field scale application light cost can be neglected as sunlight would be the source of energy.

Considering the case for MW-D-CF-algal batch study where biomass productivity was 132 $mgL^{-1}d^{-1}$, (Table 5.6) the energy produced every day per 100L of leachate was calculated based on Eq. (S2). The energy produced from algal biomass fuel per day is given below.

$$Fuel\ Energy\ = calorific\ value \times \frac{biomass\ productivity \times volume}{1000} \qquad (S2)$$

$$= 16.5\ KJg^{-1} \times \frac{132mgL^{-1}d^{-1} \times 100L}{1000\,\frac{mg}{g}} = 217.8\ KJd^{-1}$$

Fuel energy of 217.8 KJ is equivalent to 58 Wh which mean it can be used to recover some energy spent in algal reactor. In addition, calorific value of harvested biomass have greater scope for bio-diesel production and sustainable application of the developed hybrid reactor system.
